# Systematic review of dexketoprofen in acute and chronic pain

**DOI:** 10.1186/1472-6904-8-11

**Published:** 2008-10-31

**Authors:** R Andrew Moore, Jodie Barden

**Affiliations:** 1 Pain Research and Nuffield Department of Anaesthetics, University of Oxford, Level 6, West Wing, John Radcliffe Hospital, Oxford OX3 9DU, UK

## Abstract

**Background:**

Dexketoprofen, an NSAID used in the management of acute and chronic pains, is licensed in several countries but has not previously been the subjected of a systematic review. We used published and unpublished information from randomised clinical trials (RCTs) of dexketoprofen in painful conditions to assess evidence on efficacy and harm.

**Methods:**

PubMed and Cochrane Central were searched for RCTs of dexketoprofen for pain of any aetiology. Reference lists of retrieved articles and reviews were also searched. Menarini Group produced copies of published and unpublished studies (clinical trial reports). Data were abstracted into a standard form. For studies reporting results of single dose administration, the number of patients with at least 50% pain relief was derived and used to calculate the relative benefit (RB) and number-needed-to-treat (NNT) for one patient to achieve at least 50% pain relief compared with placebo.

**Results:**

Thirty-five trials were found in acute pain and chronic pain; 6,380 patients were included, 3,381 receiving dexketoprofen. Information from 16 trials (almost half the total patients) was obtained from clinical trial reports from previously unpublished trials or abstracts. Almost all of the trials were of short duration in acute conditions or recent onset pain.

All 12 randomised trials that compared dexketoprofen (any dose) with placebo found dexketoprofen to be statistically superior. Five trials in postoperative pain yielded NNTs for 12.5 mg dexketoprofen of 3.5 (2.7 to 4.9), 25 mg dexketoprofen of 3.0 (2.4 to 3.9), and 50 mg dexketoprofen of 2.1 (1.5 to 3.5). In 29/30 active comparator trials, dexketoprofen at the dose used was at least equivalent in efficacy to comparator drugs. Adverse event withdrawal rates were low in postoperative pain and somewhat higher in trials of longer duration; no serious adverse events were reported.

**Conclusion:**

Dexketoprofen was at least as effective as other NSAIDs and paracetamol/opioid combinations. While adverse event withdrawal was not different between dexketoprofen and comparator analgesics, the different conditions and comparators studies precluded any formal analysis. Exposure was limited, and no conclusions could be drawn about safety in terms of serious adverse events like gastrointestinal bleeding or cardiovascular events.

## Introduction

Racemic ketoprofen is used as an analgesic and an anti-inflammatory agent, and is one of the most potent in vitro inhibitors of prostaglandin synthesis, but is also implicated as having an association with higher risk of serious gastrointestinal bleeding events than other NSAIDs [1,2]. The analgesic effect is due to the S(+)-enantiomer (dexketoprofen), while the R(-)-enantiomer is devoid of analgesic activity [3]. Because the R(-)-enantiomer does appear to have ulcerogeneic activity, at least in the rat [3,4], the implication is that use of dexketoprofen alone should produce equivalent analgesia to double-dose ketoprofen, but at lower risk of harm. At least one case-control study in Spain appears to confirm a lower rate of serious gastrointestinal harm with dexketoprofen than ketoprofen, but with overlapping confidence intervals and small numbers of events [2]. Other authorities regard the approach of using an active enantiomer as a tromethamine salt as attractive on theoretical grounds [4].

Formulation is important, especially the use of the trometamol salt for rapid absorption [3]. In healthy volunteers absorption of dexketoprofen from dexketoprofen trometamol capsules was similar to ketoprofen, while the extent of absorption of dexketoprofen free acid was significantly lower than that for ketoprofen [5]. Dexketoprofen trometamol showed the most rapid absorption rate, with highest maximum plasma concentration and shortest time to maximum values, while ketoprofen had an intermediate absorption rate, and dexketoprofen free acid the slowest absorption rate. After repeated-dose administration of dexketoprofen trometamol, the pharmacokinetic parameters were similar to those obtained after single doses, indicating that no drug accumulation occurred [5]. Food slowed absorption of dexketoprofen, even from the trometamol salt [6].

Dexketoprofen is licensed in a number of countries around the world. Oral dexketoprofen was approved in the European Countries through a Mutual Recognition Procedure on 13th February 1998 and the injectable formulation on 25th October 2002. Dexketoprofen has not been subjected to the scrutiny of a systematic review, and not reviewed at all since preclinical and clinical development studies were reviewed over a decade ago [7]. We sought to obtain published and unpublished information from randomised clinical trials of dexketoprofen to assess the available evidence on efficacy and harm.

Systematic reviews are useful for pulling together all the studies on a topic – here randomised, double blind comparative trials of dexketoprofen in painful conditions. By assessing trial quality and validity [8,9] it is possible to eliminate trials likely to be biased, and biased trials are much more likely to over-estimate treatment effects. Accumulating many similar trials together reduces the possibility of variation in efficacy estimates because of the random play of chance, and should improve assessment of harm.

## Methods

We searched PubMed, and Cochrane Central up to October 2008 for randomised controlled trials using dexketoprofen to treat pain of any aetiology. The detailed search strategy included use of the drug name dexketoprofen anywhere in an article, together with the publication descriptor of randomised trial; this was modified for the different databases. Reference lists of retrieved articles and reviews were also searched for relevant trials. In addition, Menarini Group also produced copies of published and unpublished studies, the latter in the form of clinical trial reports.

For inclusion, trials had to be at least randomised, and use dexketoprofen to treat adult patients with pain of any origin. Trials had to have a minimum of 10 patients per treatment arm, and at least one dose of dexketoprofen given by any route of administration, at any dose, and with any comparator.

The abstracts were read, and potentially useful reports retrieved in full paper copy. No information was taken from posters or abstracts unless supplemented by details from a clinical trial report. Decisions on inclusion or exclusion of trials, assessment of trial quality and validity and all data extraction were made independently by both reviewers, with discrepancies resolved by consensus.

Methodological quality of included studies was assessed using the validated 5-point Oxford quality scale [8] utilising reporting of randomisation, blinding and withdrawals. The maximum score possible was 5 points, and no study could be included with fewer than 2 points (one for randomisation and one for blinding). Study validity was assessed using the validated Oxford Pain Validity Scale (OPVS) 16-point scale [9]. Only trials that were both randomised and double blind were used for calculation of numbers needed to treat.

Data were abstracted into a standard form. Information was extracted from the trials according to painful condition, with details of drugs, dose, route of administration, patient numbers, treatment and observation schedule, outcomes measured, and main efficacy and safety results.

For studies reporting results of single dose administration we sought to the outcome of at least 50% pain relief. For each report, mean TOTPAR (total pain relief) or SPID (summed pain intensity difference) for active and placebo groups were converted to %maxTOTPAR or %maxSPID by division into the calculated maximum value [10]. The proportion of patients in each treatment group who achieved at least 50%maxTOTPAR was calculated using verified equations [11-13]. These proportions were then converted into the number of patients achieving at least 50%maxTOTPAR by multiplying by the total number of patients in the treatment group. Information on the number of patients with at least 50%maxTOTPAR for active treatment and placebo was then used to calculate relative benefit (RB) and number needed-to-treat (NNT). Pain measures accepted for the calculation of TOTPAR or SPID were:

• 5-point categorical pain relief (PR) scales with comparable wording to "none, slight, moderate, good or complete"

• 4-point categorical pain intensity (PI) scales with comparable wording to "none, mild, moderate, severe"

• Visual analogue scales (VAS) for pain relief

• VAS for pain intensity

• 5-point categorical global scale with the wording "poor, fair, good, very good, excellent" [14]

Other measures of pain relief were abstracted where reported and appropriate. Secondary outcomes were withdrawals (all cause, lack of efficacy and adverse events) and adverse events (patients with at least one adverse event, serious adverse events, and specific adverse events). We anticipated that reporting of adverse events would vary between trials with regard to the terminology used, method of ascertainment, and categories reported (e.g. occurring in ≥ 5% of patients or where there was a statistically significant difference between treatment groups).

Guidelines for quality of reporting of meta-analyses were followed where appropriate [15]. The prior intention was to pool data where there was clinical and methodological homogeneity, with similar patients, dose, duration, outcomes, and comparators, but not where numbers of events were small, and random chance could dominate effects of treatment [16]. Homogeneity tests and funnel plots, though commonly used in meta-analysis, were not used here because they have been found to be unreliable [17,18]. Instead clinical homogeneity was examined graphically [19]. Relative benefit (or risk) and number-needed-to-treat or harm (NNT or NNH) were calculated with 95% confidence intervals. Relative benefit or risk was calculated using a fixed effects model [20], with no statistically significant difference between treatments assumed when the 95% confidence intervals included unity. We added 0.5 to treatment and comparator arms of trials in which at least one arm had no events. Number-needed-to-treat (or harm) was calculated by the method of Cook and Sackett [21] using the pooled number of observations only when there was a statistically significant difference of relative benefit or risk (where the confidence interval did not include 1). There was a prior intention to carry out sensitivity analyses for high versus low trial quality (< 3 vs ≥ 3), dose, and condition. Information would be reported with any number of patients, but not regarded unless there was a minimum of two trials or 250 patients [16].

## Results

Thirty-five trials were found in acute and chronic pain, 32 of which had reporting quality of 3/5 or better and 30 of which had OPVS score of at least 9/16 (Table 1). Ten trials had individual group sizes of 100 patients or more. The total number of patients was 6,380, of whom 3381 received dexketoprofen (Table 1). More patients were in trials of oral therapies (4,249 total, 2,270 on dexketoprofen) than trials of intramuscular or intravenous therapies (2,131 total, 1,111 on dexketoprofen). Information from 16 trials (46%) with 3,253 patients (51%) was obtained from clinical trial reports from previously unpublished trials, or trials published only as abstracts. All 16 clinical trial reports had a quality score of at least 3/5 and an OPVS score of at least 9/16. Almost all of the trials were of short duration in acute conditions, or for recent onset pain. Only two, in osteoarthritis, investigated efficacy in chronic painful conditions.

**Table 1:** Summary table of randomised trials included in the review

	**Number of:**				**Number of patients**			
			
**Pain condition**	**Studies**	**Studies with QS ≥ 3/5**	**Studies with OPVS ≥ 9/16**	**Trials of group size ≥ 100**	**In total**	**Given dexketoprofen**	**Better than placebo/total comparisons**	**At least equivalent to effective analgesic/total comparisons**
Dental pain	7	6	6	0	994	618	4/4	3/4
Postsurgical	13	11	11	2	2185	1022	7/7	11/11
Renal colic	3	3	3	3	838	526		3/3
Gynaecologic pain	2	2	1	1	350	200	1/1	2/2
Lower limb injury	1	1	1	0	122	65		1/1
Ankle sprain	1	1	1	1	209	106		1/1
Acute bone pain in cancer	1	1	1	0	115	57		1/1
Acute low back pain	5	5	5	3	1267	635		5/5
OA/RA	2	2	2	0	300	152		2/2

Total	35	32	31	10	6380	3381	12/12	29/30

QS = quality score; OPVS = Oxford Pain Validity Score; OA = osteoarthritis; RA = rheumatoid arthritis

All 12 randomised trials that compared dexketoprofen, at any dose, with placebo found dexketoprofen to be statistically superior (Table 1). More common was a comparison of dexketoprofen with an active comparator, which happened in 30 trials. In 29 of these 30 trials, dexketoprofen at the dose used was at least equivalent in efficacy to the comparator drugs with known analgesic efficacy.

### Single and multiple dose trials in dental pain

Seven randomised trials [22-29] examined the analgesic efficacy of oral dexketoprofen in 994 patients studied in the third molar extraction pain model, 618 of whom received dexketoprofen (Additional file 1). One trial was published as an abstract [29], with data taken from a clinical trial report [23]. Six of the seven trials were both randomised and double blind, and had quality scores of 4 or 5 of the maximum 5 points and had OPVS scores of at least 9/16. One open trial [27] scored only 1 out of 5.

**Figure 1:**
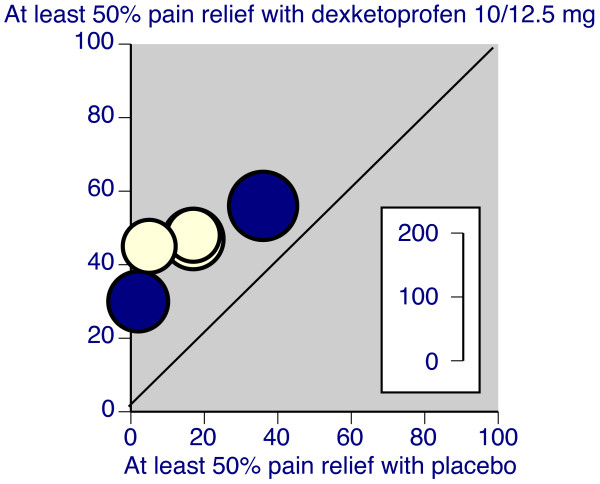
**L'Abbé plot of individual trials of dexketoprofen 10/12.5 mg compared with placebo in dental and postsurgical pain. **Inset scale shows size of trial. Light symbols = dental trials, dark symbols = postsurgical trials.

**Figure 2:**
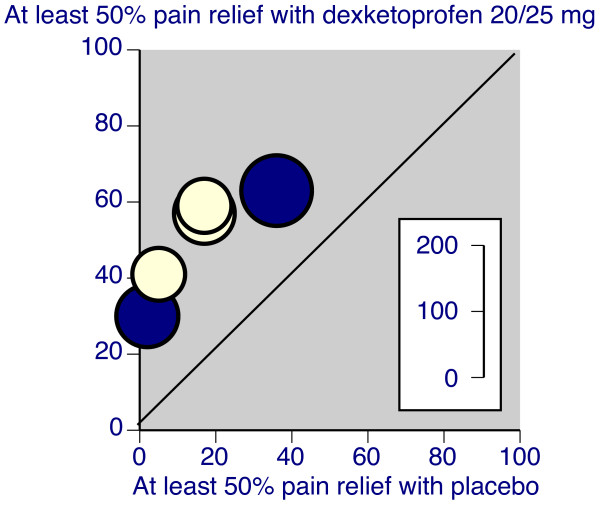
**L'Abbé plot of individual trials of dexketoprofen 20/25 mg compared with placebo in dental and postsurgical pain.** Inset scale shows size of trial. Light symbols = dental trials, dark symbols = postsurgical trials.

Three good quality trials were standard pain models reporting pain intensity or pain relief for four to six hours after the initial dose, had patients with moderate or severe pain at entry, and measured pain intensity and pain relief over six hours [24,25,28]. In these three trials dexketoprofen at doses of 10 or 12.5 mg (Figure 1), 20 or 25 mg (Figure 2), and 50 mg were all significantly superior to placebo, with NNTs for at least 50% pain relief over six hours compared with placebo of 3.0 (2.3 to 4.4), 2.6 (2.0 to 3.5), and 2.1 (1.5 to 3.5) respectively (Table 2). One trial [28] used ketoprofen 50 mg, and that was also significantly better than placebo. The one other trial that used placebo [26] reported data at eight hours, and appeared to measure pain scores after use of remedication. Despite that, dexketoprofen 25 mg was significantly better than placebo.

**Table 2:** Results of single dose trials in dental and postsurgical pain for comparison of dexketoprofen with placebo, and dexketoprofen with ketoprofen

**Dexketoprofen versus placebo**						
	**Number of**		**Percent of patients with at least 50% pain relief**			
			
**Dexketoprofen dose (mg)**	**Trials**	**Patients**	**Dexketoprofen**	**Placebo**	**Relative benefit (95% CI)**	**NNT (95% CI)**

**All trials**						
10/12.5 mg	5	462	45	17	3.4 (2.2 to 5.6)	3.5 (2.7 to 4.9)
20/25 mg	5	455	50	17	3.9 (2.4 to 6.3)	3.0 (2.4 to 3.9)
50 mg	1	67	56	8	6.7 (1.7 to 26)	2.1 (1.5 to 3.5)

**Dental**						
10/12.5 mg	3	261	47	13	3.5 (2.2 to 5.6)	3.0 (2.3 to 4.4)
20/25 mg	3	254	52	13	3.9 (2.4 to 6.3)	2.6 (2.0 to 3.5)
50 mg	1	67	56	8	6.7 (1.7 to 26)	2.1 (1.5 to 3.5)

**Postsurgical**						
10/12.5 mg	2	201	43	21	2.1 (1.4 to 3.3)	4.4 (2.8 to 9.7)
20/25 mg	2	201	47	21	2.3 (1.5 to 3.6)	3.7 (2.5 to 7.0)

**Dexketoprofen versus ketoprofen**						

	**Number of**		**Percent of patients with at least 50% pain relief**			
			
**Dexketoprofen/ketoprofen dose (mg)**	**Trials**	**Patients**	**Dexketoprofen**	**Ketoprofen**	**Relative benefit (95% CI)**	**NNT (95% CI)**

**All trials**						
12.5 vs 50	3	287	44	35	0.8 (0.5 to 1.2)	not calculated
25 vs 50	3	284	51	35	1.1 (0.7 to 1.5)	not calculated
50 vs 100	1	247	82	77	1.1 (0.9 to 1.2)	not calculated

25/50 vs 50/100	4	531	65	54	1.2 (1.1 to 1.4)	8.8 (5.1 to 33)

Dexketoprofen 12.5 mg and 25 mg were both superior to dipyrone 575 mg in the single dose phase of a multiple dose trial [22]. There was no difference between use of pre and postsurgical dexketoprofen in another trial [23,29]. The final trial [27] compared dexketoprofen 25 mg with ibuprofen 600 mg, but no interpretation could be made in this case because it included patients with mild pain which is known to desensitise pain trials.

### Single and multiple dose trials in postsurgical pain

Thirteen randomised trials [30-43] examined the analgesic efficacy of dexketoprofen in 2135 patients studied in postsurgical pain, 997 of whom received dexketoprofen (Additional file 2). One trial was published as an abstract [31] with data taken from a clinical trial report [42]. Twelve of the 13 trials were both randomised and double blind, and 11 had quality scores of three or more of the maximum 5 points and at least 9 on an OPVS (Table 1). Eight trials (1212 patients) used oral dexketoprofen and four (923 patients) intramuscular or intravenous dexketoprofen. Eight of the 13 trials were in major orthopaedic surgery (mainly knee and hip surgery), the others involving arthroscopy, bunionectomy, hernias, abdominal hysterectomy, and abdominal surgery.

Two good quality trials were standard pain models reporting pain intensity or pain relief for four to six hours after the initial dose, had patients with moderate or severe pain at entry, and measured pain intensity and pain relief over six hours [32,34]. In these trials oral dexketoprofen at doses of 10 or 12.5 mg (Figure 1) and 20 or 25 mg (Figure 2) were significantly superior to placebo, with NNTs for at least 50% pain relief over six hours compared with placebo of 4.4 (2.8 to 9.7) and 3.7 (2.5 to 7.0) respectively (Table 2). Four of the nine oral trials used placebo, and in these dexketoprofen was significantly better than placebo on at least one measure in three trials [34,39,43], but not in the fourth [32]. Ketoprofen 50 mg was not significantly better than placebo in the two trials that used it [32,34].

Where there was an active comparator, dexketoprofen 25 mg appeared to be equivalent to tramadol 50 mg [42,33], diclofenac 50 mg [36], and paracetamol 500 mg plus codeine 22.5 mg [38]. Three trials compared dexketoprofen 25 mg with ketoprofen 50 mg; while there was no difference in one small trial [41], ketoprofen appeared to be less effective in two others [32,34].

Two trials [35,40] used intramuscular administration of dexketoprofen at doses of 25 mg or 50 mg twice a day, and two [30,37] intravenous administration of 50 mg three times a day, or 50 mg twice a day. Time intervals between doses were 6–8 h and 12 hours in the different studies. Three [35,37,40] made a comparison with placebo, and in all three doses of dexketoprofen were significantly better than placebo on at least one measure of efficacy. All four trials had an active comparator, and dexketoprofen at the dose studied was at least as effective as ketoprofen 100 mg [30,40], tramadol 100 mg [37], and diclofenac 75 mg twice a day [35]. There was a suggestion of somewhat better efficacy between three and eight hours, and lower morphine requirements, than diclofenac 75 mg twice a day [35].

### Overall results of single dose dexketoprofen in acute pain, and comparison with ketoprofen

Combining three third molar extraction and two postsurgical trials (Table 2) gave NNTs for at least 50% pain relief for 12.5 mg dexketoprofen of 3.5 (2.7 to 4.9), 25 mg dexketoprofen of 3.0 (2.4 to 3.9), and 50 mg dexketoprofen of 2.1 (1.5 to 3.5). The overlapping confidence intervals and formal testing [44] for difference between NNTs showed no statistical difference between 12.5 mg and 25 mg doses.

**Figure 3:**
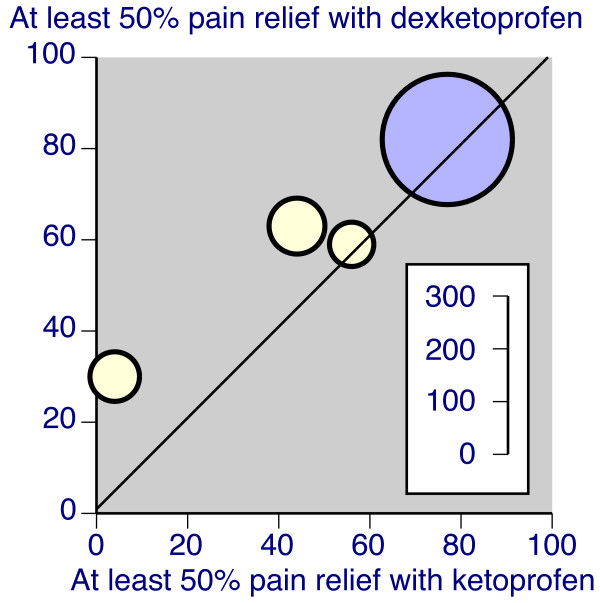
**L'Abbé plot of individual trials of dexketoprofen compared with double dose of ketoprofen in dental and postsurgical pain.** Inset scale shows size of trial. Light symbols = 25 mg vs 50 mg, dark symbols = 50 mg vs 100 mg.

Several trials used both dexketoprofen and ketoprofen. Table 2 also shows the comparisons between 12.5 mg and 25 mg dexketoprofen and 50 mg ketoprofen, and 50 mg dexketoprofen and 100 mg ketoprofen. While the proportion of patients achieving at least 50% pain relief was consistently higher with dexketoprofen, this did not reach statistical significance with any comparison. However, when 25 mg or 50 mg dexketoprofen were compared with 50 mg or 100 mg ketoprofen (that is, double the dose, Figure 3), statistical significance was achieved, with a number needed to treat of 8.8 (5.1 to 33). That means that for every nine persons treated with 25 mg or 50 mg dexketoprofen, one more would have at least 50% pain relief than if the same nine patients were treated with ketoprofen 50 mg or 100 mg.

### Single dose trials in pain of renal colic

Three randomised trials [45-47] examined the analgesic efficacy of dexketoprofen 25 mg and 50 mg intramuscularly, and 25 mg and 50 mg intravenously, in 838 patients studied in pain of renal colic, 526 of whom received dexketoprofen (Additional file 3). All of the trials were both randomised and double blind, all had quality scores of three or more of the maximum 5 points and at least 9 points on an OPVS. One trial [45] used intramuscular dexketoprofen and two [46,47] intravenous dexketoprofen.

None of the trials had a placebo control, and all examined efficacy over six hours after a single dose n pain of moderate or severe intensity. Intramuscular dexketoprofen 25 mg and 50 mg were indistinguishable from intramuscular dipyrone 2000 mg [45]. Intravenous dexketoprofen 25 mg or 50 mg were indistinguishable from intravenous dipyrone 2000 mg or more [46], and intravenous dexketoprofen 50 mg was indistinguishable from intravenous ketoprofen 100 mg [47].

### Multiple dose trials in acute low back pain

Five trials [48-53] examined short-term use of dexketoprofen in acute low back pain, generally over about a week (Additional file 4); one was published in German [50], but data were taken from a clinical trial report [49]. All of the trials were both randomised and double blind, all had quality scores of three or more of the maximum 5 points and at least 9 points on an OPVS. One shorter trial compared 50 mg twice-daily intramuscular dexketoprofen with 75 mg diclofenac in almost 400 patients [48]. Four oral comparisons of dexketoprofen 25 mg three times daily over 4–7 days in patients with pain of acute onset back pain of at least moderate severity showed similar efficacy to diclofenac 150 mg daily [51], tramadol 150 mg daily [49,52], and paracetamol 800 mg plus dextropropoxyphene 60 mg daily [53].

### Single and multiple dose trials in other acute painful conditions

Five randomised trials [54-58] have examined the analgesic efficacy in other acute painful conditions in 796 patients, 428 of whom received oral dexketoprofen, mainly at 25 mg (Additional file 5). All of the trials were both randomised and double blind, all had quality scores of three or more of the maximum 5 points and at least 9 points on an OPVS. Only one trial [54] was placebo controlled, and looked at efficacy of 12.5 mg and 25 mg of dexketoprofen in comparison with 50 mg ketoprofen in 52 women with dysmenorrhoea; all three active treatments were superior to placebo, but not different one from another.

Dexketoprofen 25 mg orally was found to be superior to injections of mepivacaine into the uterine cervix in producing significantly lower pain scores over 30–120 minutes after hysteroscopy [55] as well as being better than 50 mg diclofenac for lower limb injury between 15 and 60 minutes [56]. Over four days there was no difference between three times daily ketoprofen 25 mg or paracetamol 500 mg plus codeine 60 mg in the treatment of ankle sprains [57]. In patients with cancer who developed bone cancer pain of at least moderate intensity, and who had not previously been treated with a continuous regimen of opioids or NSAIDs in the previous 15 days, there was no difference between 25 mg dexketoprofen and 10 mg ketorolac over seven days [58].

### Multiple dose trials in arthritis

Two trials tested dexketoprofen 25 mg three times a day against ketoprofen 150 mg daily and diclofenac 150 mg daily in patients with established arthritis [59,60](Additional file 6). Both trials were randomised and double blind, all had quality scores of three or more of the maximum 5 points and at least 9 points on an OPVS. The trials had a flare design in which patients discontinued previous treatment. Over two or three weeks of treatment there were no differences between dexketoprofen and diclofenac at these doses [60], though dexketoprofen 75 mg daily was superior to ketoprofen 150 mg daily [59].

### Overall comparison between dexketoprofen and ketoprofen

The main comparisons between dexketoprofen and ketoprofen occurred within the dental trials and those in postsurgical pain. There were three other comparisons. One comparison of intravenous administration in renal colic showed no difference between dexketoprofen 50 mg and ketoprofen 100 mg [47]. Of the two oral comparisons there was no difference between dexketoprofen 12.5 mg or 25 mg and ketoprofen 50 mg [54], while the one comparison between 25 mg dexketoprofen with 50 mg ketoprofen in arthritis showed better results for dexketoprofen [59].

### Adverse events

Additional files 1, 2, 3, 4, 5, 6 records adverse events reported in the trials, in terms of the numbers of patients reporting at least one adverse event, all cause withdrawals, and withdrawal due to an adverse event. Adverse event reporting was generally poor. Because trials varied from single dose to three weeks duration, with different routes of administration, drug doses, comparators, and condition, sensible analysis of adverse events were not possible. Because adverse event withdrawal is a significant event, and attempt was made to examine adverse event withdrawal rates in trials where at least two doses of drug were given. Because the rate of adverse event withdrawals is likely to be a function of the number of doses given, these were split by relatively shorted duration studies predominantly less than two days (dental and postsurgical pain) and relatively longer studies predominantly more two days or longer (acute painful conditions, back pain, and arthritis) (Table 3).

**Table 3:** Adverse event withdrawal rates in trials where at least two doses of drug were given

	**Dental and postsurgical pain**		**Other acute, back pain, arthritis**	
	
**Drug**	**Number of patients**	**Adverse event withdrawal (%)**	**Number of patients**	**Adverse event withdrawal (%)**
Placebo	236	2.5	no data	
Dexketoprofen	652	1.8	844	3.2
Ketoprofen	301	1.3	152	7.9
Diclofenac	80	0.0	272	3.7
Tramadol	72	1.4	247	9.7
Paracetamol + opioid	100	0.0	167	1.2

The choice of two doses was simply because withdrawal is not really an option after a single dose and is unlikely to be recorded in the same was as in multiple dose studies.

In both comparisons dexketoprofen (all doses) provided the about half the total number of patients (Table 3). Adverse event withdrawal rates were low, at about 2% or below in dental and postsurgical pain, and somewhat higher in trials of longer duration. The adverse event withdrawal rate for dexketoprofen was not out of line with other drugs, though limited numbers prevented any firm conclusions, and statistical tests were not deemed sensible.

No serious adverse events, like gastrointestinal bleeding, myocardial infarction, or death, were reported in any trial.

## Discussion

This review found reports of 34 randomised trials of dexketoprofen, predominantly of sufficiently high reporting quality to avoid bias [9,61]. To be comprehensive any randomised trial was included, but only higher quality trials (randomised, double blind) were used to calculate NNTs. Almost half the trials and just over half the patients (51%) were in trials that had not previously been published in full, and so this review doubles the amount of information previously available on dexketoprofen. Significant numbers of otherwise unpublished pain trials have been found before in systematic reviews [62,63].

Nearly all trials appeared to be valid as judged by quality scores and OPVS scores. The two arthritis trials, at three weeks, were considerably shorter than the current norm in arthritis trials, which now is 6–12 weeks. The trials tended to be relatively small, with an average of 190 patients split between several treatment groups, and while they were sufficient to yield statistical results regarding the direction of any effect, they were not individually large enough to comment sensibly on its magnitude [16]. While 10 trials had group sizes of at least 100 patients, these were spread throughout the different conditions studied (Table 1).

The small size and generally short duration of the trials limits transfer of knowledge to clinical practice. The trials tell us about whether dexketoprofen is an analgesic. They do not tell us how best to use it in any particular painful condition.

Meta-analysis of all trials was not possible because of the differences between them in terms of painful condition being treated, dose and route of administration of dexketoprofen, duration of therapy, and outcomes reported. Vote counting only was possible, and this showed that all 12 trials with a placebo comparison showed dexketoprofen to be better than placebo, and that 29/30 trials showed dexketoprofen to be at least equivalent to an active comparator of known analgesic efficacy (Table 1).

The one area where meta-analysis was possible was that of single dose oral administration in dental and postsurgical pain (Table 2). Based on limited data there appeared to be a dose-response, with better (lower) NNTs with higher doses of dexketoprofen. The best general comparison with other analgesics probably comes from the dental pain model, because these trials are consistently conducted in very similar patients, using similar methods and outcomes, and tried and tested methods [64,65]. The NNT for dexketoprofen compared with placebo for at least 50% pain relief over 4–6 hours was 2.6, comparable to ibuprofen 200–600 mg (NNTs 2.2–2.8) and diclofenac 50 mg (NNT 2.1), and better than paracetamol 1000 mg (NNT 3.7) [64]. Limited numbers of patients for some of these drugs and doses make it invidious to push these comparisons too far, but at least it can be said that oral dexketoprofen 25 mg is an effective analgesic according to present standards. As yet we do not have sufficient or consistent information across systematic reviews of single dose analgesics to make comparisons of duration of analgesia (median time to remedication, or percentage of patients remedicating in a fixed time, for instance), though this would be useful additional information [66].

There available evidence is that analgesia with dexketoprofen is equivalent to analgesia obtained with double the dose of ketoprofen. In single doses in acute pain, there is a hint even of superior analgesia than double dose ketoprofen (Figure 3, Table 2), and there is at least equivalence in three other trials.

Again, the varied nature of the studies precluded any formal meta-analysis of adverse events. What could be done was a descriptive analysis of adverse event withdrawals in trials with at least two doses of dexketoprofen. The split by relatively short term studies in dental and postsurgical pain, and somewhat longer studies in acute pain, back pain, and arthritis (Table 3) appeared to make sense, as withdrawal rates tended to be somewhat higher in the longer duration studies. Dexketoprofen adverse event withdrawals were not higher than other effective analgesics, based on the limited data available.

No conclusions could be drawn about serious adverse events like serious gastrointestinal bleeding, cardiovascular events, or mortality. Gastrointestinal bleeding and cardiovascular events tend to occur at a rate of about 1% a year in randomised trials in arthritis [67]. Trials of dexketoprofen lasted only three weeks with arthritis, and barely a week with most trials. In that circumstance, the rate of a serious adverse event would be expected in about 1 in 5,000 patients (1 in 100 multiplied by 50), and only 3,200 patients were in trials other than dental or postsurgical pain. Additionally, a number of those trials were in patients substantially younger than those in arthritis trials, with substantially lower baseline risk, decreasing the potential risk even lower than 1 in 5,000. The absence of serious events should not, therefore, be taken as an absence of risk, because the quantity, type and duration of studies precludes any such conclusion.

## Conclusion

This review doubles the amount of information available concerning analgesic efficacy of dexketoprofen. That efficacy was apparent in single dose in dental and postsurgical pain, where NNTs for at least 50% pain relief over 4–6 hours compared with placebo were similar to other effective analgesics. In vote-counting, dexketoprofen was at least as effective as other analgesics in 29/30 trials. While adverse event withdrawal was not different between dexketoprofen and comparator analgesics, the different conditions and comparators studies precluded any formal analysis. The amount of exposure was limited, and no conclusions could be drawn about safety in terms of serious adverse events like gastrointestinal bleeding or cardiovascular events.

## Competing interests

RAM has received research grants, consulting, or lecture fees from pharmaceutical companies, government sources, and charities. Neither author has any direct stock holding in any pharmaceutical company.

## Authors' contributions

RAM the original concept, planning the study, searching, data extraction, writing, analysis, and preparing a manuscript; JB was involved with searching, data extraction, writing, analysis, and preparing a manuscript.

## Pre-publication history

The pre-publication history for this paper can be accessed here:



## Supplementary Material

Additional file 1 **Trials of oral dexktoprofen in pain after third molar extraction pain.** The file contains information on each included study, with reference, quality score, design, treatments, main results, and comments. Click here for file

Additional file 2 **Trials of oral and injected dexktoprofen in pain after surgery.** The file contains information on each included study, with reference, quality score, design, treatments, main results, and comments. Click here for file

Additional file 3 **Trials of injected dexktoprofen in pain of renal colic.** The file contains information on each included study, with reference, quality score, design, treatments, main results, and comments. Click here for file

Additional file 4 **Trials of intramuscular and oral dexktoprofen in acute back pain.** The file contains information on each included study, with reference, quality score, design, treatments, main results, and comments. Click here for file

Additional file 5 **Trials of oral dexktoprofen in gynaecological and other acute painful conditions.** The file contains information on each included study, with reference, quality score, design, treatments, main results, and comments. Click here for file

Additional file 6 **Trials of oral dexktoprofen in arthritis. **The file contains information on each included study, with reference, quality score, design, treatments, main results, and comments. Click here for file
