# Supplementary material for: Systematic review of dexketoprofen in acute and chronic pain
Source: BMC Clin Pharmacol. 2008 Oct 31;8:11. doi: 10.1186/1472-6904-8-11 (PMC2585070; doi:10.1186/1472-6904-8-11)
Supplement: Additional file 1 — Trials of oral dexktoprofen in pain after third molar extraction pain. The file contains information on each included study, with reference, quality score, design, treatments, main results, and comments. [file 1472-6904-8-11-S1.pdf]

Additional file 1: Trials of oral dexktoprofen in pain after third molar extraction pain

Reference	Methods	Details	Dosing regimen	Outcomes	Efficacy Results	Remedication, exclusions, and adverse events	Safety results	Quality score
Gay et al. Analgesic Efficacy of Low Doses of Dexketoprofen in the Dental Pain Model. Clin Drug Invest 1996; 11: 320-330	RCT, DB, single oral dose, parallel groups, LA, 12 hr analgesic washout<br><br>Assessed at baseline, 10, 20, 30 and 45 mins, and 1, 1.5, 2, 3, 4, 5, 6 hrs<br><br>Medication administered when pain was of moderate to severe intensity and within 3 hrs of surgery	Third molar extraction<br><br>N= 206<br><br>2 centres (Barcelona and Spain)	Dexketoprofen tromethamine 5mg<br>N= 41<br><br>Dexketoprofen tromethamine 10mg<br>N= 42<br><br>Dexketoprofen tromethamine 20mg N= 41<br><br>Ibuprofen 400mg<br>N= 41<br><br>Placebo<br>N= 41	Pain intensity<br>100mm VAS<br><br>Pain intensity<br>4-pt VRS (1-absent, 2- mild, 3 - moderate, 4 - severe)<br><br>Pain relief<br>5-pt VRS (0 - nil, 1 - slight, 2 - moderate, 3 - considerable, 4 - complete)<br><br>Time to remedication<br><br>Patient global<br>4-pt VRS (useless, mediocre, good, excellent)	Dexketoprofen tromethamine 5mg<br>SPID6 (VAS) 77.9 ± 127.2<br>TOTPAR6 9.8 ± 6.4<br>Global good/excellent 59%<br>Time to onset 2.8 ± 2.6<br>Time to remedication 300.4 ± 96.7<br>No remedicating 14<br><br>Dexketoprofen tromethamine 10mg<br>SPID6 96.7 ± 158.8<br>TOTPAR6 10.5 ± 6.7<br>Global good/excellent 68%<br>Time to onset 1.7 ± 2.3<br>Time to remedication 289.6 ± 93.3<br>No remedicating 20<br><br>Dexketoprofen tromethamine 20mg<br>SPID6 109.8 ± 129.3<br>TOTPAR6 11.3 ± 6.4<br>Global good/excellent 68%<br>Time to onset 0.9 ± 1.3<br>Time to remedication 302.3 ± 85.7<br>No remedicating 18<br><br>ibuprofen 400mg<br>SPID6 148.7 ± 153.6<br>TOTPAR6 13.6 ± 7.5<br>Global good/excellent 78%<br>Time to onset 2.1 ± 2.3<br>Time to remedication 302.6 ± 106.1<br>No remedicating 11<br><br>Placebo SPID6 (VAS) -19.5 ± 141.7<br>TOTPAR6 5.2 ± 5.8<br>Global good/excellent 29%<br>Time to onset 4.0 ± 2.6<br>Time to remedication 218.9 ± 122.7<br>No remedicating 26<br><br>All three dexketoprofen doses and ibuprofen significantly better than placebo	Remedication permitted, LOCF for patients remedicating 1hr or more into study<br><br>2 patients excluded from ITT analysis for remedicating within 1 hr<br><br>A total of 17 patients reported 27 adverse events, all were minor and no patient withdrew as a result	Dexketoprofen tromethamine 5mg<br>No with >1 AE 3<br>All cause withdrawals 0<br>AE withdrawals 0<br><br>Dexketoprofen tromethamine 10mg<br>No with >1 AE 2<br>All cause withdrawals 0<br>AE withdrawals 0<br><br>Dexketoprofen tromethamine 20mg<br>No with >1 AE 5<br>All cause withdrawals 0<br>AE withdrawals 0<br><br>Ibuprofen 400mg<br>No with >1 AE 3<br>All cause withdrawals 0<br>AE withdrawals 0<br><br>Placebo<br>No with >1 AE 4<br>All cause withdrawals 2<br>AE withdrawals 0	R 1<br>DB 2<br>WD 1<br><br>Total = 4<br><br>OPVS = 13/16

McGurk et al. Clinical comparison of dexketoprofen trometamol, ketoprofen, and placebo in postoperative dental pain. J Clin Pharmacol 1998; 38 (Suppl): 46S-54S	RCT, DB, single oral dose, parallel groups, LA, 12 hr analgesic washout<br><br>Assessed at baseline, 10, 20, 30 and 45 mins, and 1, 1.5, 2, 3, 4, 5, 6 hrs<br><br>Medication administered when pain was of moderate to severe intensity and within 3 hrs of surgery	Third molar extraction<br><br>N= 210<br><br>10 centres in the UK, 1 centre in Germany	Dexketoprofen trometamol 12.5mg<br>N= 44<br><br>Dexketoprofen trometamol 25mg<br>N= 41<br><br>Dexketoprofen trometamol 50mg<br>N= 43<br><br>Ketoprofen 50 mg (racemic)<br>N= 43<br><br>Placebo<br>N= 39	Pain intensity<br>100mm VAS<br><br>Pain intensity<br>4-pt VRS (1-absent, 2- mild, 3 - moderate, 4 - severe)<br><br>Pain relief<br>5-pt VRS (0 - nil, 1 - slight, 2 - moderate, 3 - considerable, 4 - complete)<br><br>Patient assessment of whether pain reduced by 50% from baseline<br><br>Time to remedication<br><br>Patient global<br>4-pt VRS (ineffective, mediocre, good, excellent)	Dexketoprofen trometamol 12.5mg<br>SPID6 7.07 ± 16.44<br>TOTPAR6 10.2 ± 7.27<br>Global good/excellent 58%<br>Time to max PID 2.0 ± 1.5<br>Time to remedication 4.9 ± 1.6<br>No remedicating 41%<br><br>Dexketoprofen trometamol 25mg<br>SPID6 4.84 ± 4.4<br>TOTPAR6 12.61 ± 6.13<br>Global good/excellent 65%<br>Time to max PID 1.8 ± 1.2<br>Time to remedication 5.3 ± 1.3<br>No remedicating 27%<br><br>Dexketoprofen trometamol 50mg<br>SPID6 4.52 ± 4.78<br>TOTPAR6 12.3 ± 6.7<br>Global good/excellent 78%<br>Time to max PID 2.5 ± 1.8<br>Time to remedication 5.4 ± 1.1<br>No remedicating 24%<br>Ketoprofen (racemic)<br>SPID6 4.16 ± 4.62<br>TOTPAR6 12.16 ± 6.17<br>Global good/excellent 60%<br>Time to max PID 2.5 ± 1.9<br>Time to remedication 5.5 ± 1.4<br>No remedicating 15%<br><br>Placebo<br>SPID6 -1.92 ± 4.79<br>TOTPAR6 3.23 ± 4.77<br>Global good/excellent 12%<br>Time to max PID 1.1 ± 1.6<br>Time to remedication 3.6 ± 2.0<br>No remedicating 79%<br><br>All active treatments significantly better than placebo	Remedication permitted, LOCF for patients remedicating 1hr or more into study, patients remedicating within 1 hr were excluded from analyses<br><br>10 patients were excluded; 3 due to remedicating within 1 hr and 7 because they failed to return the patient diary card<br><br>A total of 35 events were reported by 28 patients, there were no statistically significant differences between groups and the majority were mild, 2 patients withdrew as a results of adverse events	Dexketoprofen trometamol 12.5mg<br>No with >1 AE 4<br>AE withdrawals 0<br><br>Dexketoprofen trometamol 25mg<br>No with >1 AE 4<br>AE withdrawals 0<br><br>Dexketoprofen trometamol 50mg<br>No with >1 AE 7<br>AE withdrawals 0<br><br>Ketoprofen (racemic)<br>No with >1 AE 5<br>AE withdrawals 1<br><br>Placebo<br>No with >1 AE 8<br>AE withdrawals 1	R 2<br>DB 2<br>WD 1<br><br>Total = 5<br><br>OPVS = 13/16
Bagán et al. Clinical comparison of dexketoprofen trometamol and dipyrone in postoperative dental pain. J Clin Pharmacol 1998; 38 (Suppl): 55S-64S	RCT, DB, multiple oral dose, parallel groups, LA, 12 hr analgesic washout<br><br>Single-dose phase; assessed at baseline, 15, and 30 mins, and 1, 2, 3, 4, 5, 6 hrs<br><br>Multi-dose phase; assessed immediately before and 2 hr after each dose<br><br>Medication administered when pain was of moderate to severe intensity and within 3 hrs of surgery<br><br>Repeat doses (2 to 12) could be taken at least 6 hrs apart, more than 4 times a day for 3 days	Third molar extraction<br><br>N= 125<br><br>4 centres in Spain	Dexketoprofen trometamol 12.5mg<br>N= 38<br><br>Dexketoprofen trometamol 25mg<br>N= 42<br><br>Dipyrone 575mg<br>N= 40	Pain intensity<br>100mm VAS<br><br>Pain intensity<br>4-pt VRS (0 - no pain to 3 - severe pain)<br><br>Pain relief<br>5-pt VRS (0 - no relief to 4 - complete relief)<br><br>Patient global<br>4-pt VRS (poor, fair, good, excellent)<br><br>Time to remedication	Dexketoprofen trometamol 12.5mg<br>SPID6 4.8 ± 3.6<br>TOTPAR6 10.7 ± 6.6<br>Global good/excellent 82%<br>Time to max PID 108.6 ± 90.4<br>Time to remedication 335.2 ± 57.5<br>No remedicating 12<br><br>Dexketoprofen trometamol 25mg<br>SPID6 5.3 ± 4.1<br>TOTPAR6 13 ± 5.8<br>Global good/excellent 90%<br>Time to max PID 135.4 ± 96.3<br>Time to remedication 333.7 ± 69.1<br>No remedicating 8<br><br>Dipyrone 575mg<br>SPID6 3.5 ± 4.6<br>TOTPAR6 8.5 ± 8.2<br>Global good/excellent 44%<br>Time to max PID 73.1 ± 72.1<br>Time to remedication 301.1 ± 94.7<br>No remedicating 19<br><br>Both dexketoprofen doses significantly better	Remedication permitted, LOCF (pain intensity) and 'none' (pain relief) for patients withdrawing 1hr or more into the study, patients remedicating within 1 hr were excluded from analyses. Patients only included in the repeated-dose phase if they did not remedicate within the first 6 hrs. Patients remedicating at any point during the repeated dose phase were withdrawn<br><br>5 patients were excluded from single dose phase; 1 patient failed to take study medication, 1 patient remedicated within 1 hr, 3 patients reported only mild baseline pain<br><br>13 patients reported a total of 18 adverse events, there were no adverse events leading to withdrawal, does not state if results are from single-dose phase only or both phases	Dexketoprofen trometamol 12.5mg<br>No with >1 AE 8<br>AE withdrawals 0<br><br>Dexketoprofen trometamol 25mg<br>No with >1 AE 7<br>AE withdrawals 0<br><br>Dipyrone 575mg<br>No with >1 AE 3<br>AE withdrawals 0	R 1<br>DB 2<br>WD 1<br><br>Total = 5<br><br>OPVS = 13/16

Jackson et al. Double-blind, randomised, placebo-controlled trial comparing rofecoxib with dexketoprofen trometamol in surgical dentistry. Br J Anaesth 2004; 92:675-680.	RCT, DB, single oral dose, parallel groups, LA, 12 hr analgesic washout<br><br>Assessed at baseline, 15, 30, and 45 mins, and 1, 2, 3, 4, 5, 6, 7 and 24 hrs<br><br>Medication administered when pain was of moderate to severe intensity and within 4 hrs of surgery	Third molar extraction<br><br>N= 120<br><br>1 centre in Scotland	Dexketoprofen trometamol 25mg<br>N= 42<br><br>Rofecoxib 50mg<br>N= 37<br><br>Placebo<br>N= 43	Pain intensity<br>100mm VAS<br><br>Pain intensity<br>4-pt VRS (1 - absent, 2 - mild, 3 - moderate, 4 - severe)<br><br>Pain relief<br>100mm VAS<br><br>Pain relief<br>5-pt VRS (1 - none, 2 - a little, 3 - some, 4 - a lot, 5 - complete)<br><br>Patient global<br>5-pt VRS (1 - poor, 5 - excellent)	Dexketoprofen trometamol 25mg<br>SPID8 6.9 ± 3.9<br>TOTPAR8 463.9 ± 169.8<br>Global8 good/excellent 3 (2 - 4)<br>Time to remedication 398 (251 - 630)<br>No remedicating 35<br><br>Rofecoxib 50mg<br>SPID8 8.128 ± 3.86<br>TOTPAR8 502.4 ± 163.1<br>Global8 good/excellent 4 (3 - 4)<br>Time to remedication 1440 (425 - 1440)<br>No remedicating 15<br><br>Placebo<br>SPID8 4.25 ± 4.96<br>TOTPAR8 361.5 ± 182.8<br>Global8 good/excellent 2 (1-3)<br>Time to remedication 150 (88 - 256)<br>No remedicating 36<br><br>Both active drugs significantly better than	Remedication permitted, does not state how missing values handled only that ANOVA and Bonferroni's correction for multiple comparisons were applied<br><br>7 patients were excluded; 4 patients failed to develop sufficient pain, no data were available for 2 patients, 1 patient was not dosed due to severe nausea<br><br>15 patients reported 20 adverse events, there were no adverse events leading to withdrawal	Dexketoprofen trometamol 25mg<br>No with >1 AE 5<br>All cause withdrawals 0<br>AE withdrawals 0<br><br>Rofecoxib 50mg<br>No with >1 AE 6<br>All cause withdrawals 0<br>AE withdrawals 0<br><br>Placebo<br>No with >1 AE 4<br>All cause withdrawals 0<br>AE withdrawals 0	R 1<br>DB 2<br>WD 1<br><br>Total = 4<br><br>OPVS = 13/16
Jiménez-Martínez et al. Estudio de la eficacia analgésica del Dexketoprofeno trometamol 25 mg vs Ibuprofeno 600 mg tras su administración oral en pacientes sometidos a una intervención quirúrgica oral. Med Oral 2004; 9: 138-48.	RCT, multiple oral dose, parallel groups, 24 hr analgesic washout<br><br>Assessed at baseline, 1, 6 and 8 hrs after drug administration<br><br>Medication administered 2 hrs after surgery (unless pain occurred sooner although 26 patients didn't take until after 2 hrs) and then every 8 hrs; states that pain presented by patient after surgery but before treatment was used as a reference, results describe mild pain so baseline pain not moderate to severe in intensity	Third molar extraction<br><br>N= 93<br><br>1 centre in Spain	Dexketoprofen trometamol 25mg<br>N= 47<br><br>Ibuprofen 600mg<br>N= 46	Pain intensity<br>100mm VAS<br><br>Pain intensity<br>VRS	Not reported<br><br>Impossible to interpret results, as differences in no or mild pain between groups	Not reported	Not reported	R 1<br>DB 0<br>WD 0<br><br>Total = 1<br><br>OPVS = 7/16

Harrison F. Dental Pain study of dexketoprofen 12.5 mg and 25 mg versus placebo. (data from clinical trial report)	RCT, DB, single oral dose, parallel groups, LA, 12 hr analgesic washout<br><br>Assessed at baseline, 10, 20, 30 and 45 mins, and 1, 1.5, 2, 3, 4, 5 and 6 hrs<br><br>Medication administered when pain was of moderate to severe intensity and within 3 hrs of surgery	Third molar extraction<br><br>N= 141<br><br>6 centres in Germany	Dexketoprofen tromethamine 12.5 mg<br>N= 49<br><br>Dexketoprofen tromethamine 25 mg<br>N= 46<br><br>Placebo<br>N= 46	Pain intensity<br>100mm VAS<br><br>Pain intensity<br>4-pt VRS (absent, mild, moderate, severe)<br><br>Pain relief<br>5-pt VRS (no pain relief, slight pain relief, moderate pain relief, considerable pain relief, complete pain relief)<br><br>Percentage pain relief<br><br>Patient global<br>4-pt VRS (null, mediocre, good, excellent)	Dexketoprofen tromethamine 12.5 mg<br>SPID6 3.97<br>TOTPAR6 10.6<br>Global good/excellent 27<br>Time to max PID 2.02<br>Time to remedication 5.4<br>No remedicating<br><br>Dexketoprofen tromethamine 25 mg<br>SPID6 5.15<br>TOTPAR6 12.35<br>Global good/excellent 28<br>Time to max PID 2.39<br>Time to remedication<br>No remedicating<br><br>Placebo<br>SPID6 0.28<br>TOTPAR6 5.18<br>Global good/excellent 9<br>Time to max PID 1.77<br>Time to remedication 4.33<br>No remedicating 5.71<br><br>Both dexketoprofen doses significantly better than placebo	4 patients were excluded from ITT analyses as they remedicated within the 1st hr	Dexketoprofen tromethamine 12.5 mg<br>No with >1 AE 6<br>All cause withdrawals 16<br>AE withdrawals 0<br><br>Dexketoprofen tromethamine 25 mg<br>No with >1 AE 7<br>All cause withdrawals 9<br>AE withdrawals 1<br><br>Placebo<br>No with >1 AE 6<br>All cause withdrawals 21<br>AE withdrawals 0	R 2<br>DB 2<br>WD 1<br><br>Total = 5<br><br>OPVS = 13/16
Munoz et al. Preoperative dexketoprofen tromatol for the prevention of postoperative dental pain: a randomized double-blind clinical study. Methods and Findings in Experimental and Clinical Pharmacology 1998; 20 (Suppl A):69. (abstract)<br>Data from clinical trial report [Berini et al. Clinical trial to assess the analgesic efficacy of a single oral administration of LM-1158.TRIS in the prevention of post-operative pain. Clinical trial report 1999]	RCT, DB, DD, single oral dose, parallel groups,<br><br>Assessed at baseline (30 mins after surgery), 10, 20, 30 and 45 mins, and 1, 1.5, 2, 3, 4, 5, 6, 7 and 8 hrs<br><br>Medication administered either 30 mins pre-surgery or 30 mins post surgery	Third molar extraction<br><br>N= 102<br><br>1 centre in Spain	Dexketoprofen trometamol 25mg pre-surgery<br>N= 51<br><br>Dexketoprofen trometamol 25mg post-surgery<br>N= 51	Pain intensity<br>100mm VAS<br><br>Pain intensity<br>4-pt VRS (zero, mild, moderate, severe)<br><br>Patient global<br>4-pt VRS (null, mediocre, good, excellent)<br><br>Time to remedication	Dexketoprofen trometamol 25mg pre-surgery<br>SPID8 236.21 ±167<br>SPID4 67.44 ± 63.14)<br>Global good/excellent 34<br>Time to min pain intensity 10.21 ± 30.23<br>Time to remedication 282.18 ± 82.6<br>No remedicating 17<br><br>Dexketoprofen trometamol 25mg post-surgery<br>SPID8 196.69 ± 155.35<br>SPID4 68.93 ± 56.1<br>Global good/excellent 42<br>Time to min pain intensity 21.25 ± 38.68<br>Time to remedication 339 ± 84.7<br>No remedicating 14<br><br>No significant differences	Remedication permitted, patients remedicating within 1 hr were excluded from analyses, after 1 hr LOCF used to input missing values<br><br>3 adverse events were reported, all were of mild intensity, no serious adverse events were reported<br><br>4 patients were excluded; 1 patient did not require surgery, 1 patient was <18 yrs of age, 1 patient underwent surgery for >45 mins, and there was an error in treatment administration for 1 patient	Dexketoprofen trometamol 25mg pre-surgery<br>No with >1 AE 1<br>All cause withdrawals<br>AE withdrawals 0<br><br>Dexketoprofen trometamol 25mg post-surgery<br>No with >1 AE 2<br>All cause withdrawals<br>AE withdrawals 0	R 2<br>DB 2<br>WD 1<br><br>Total = 5<br><br>OPVS = 13/16

Abbreviations: RCT = randomised controlled trial; R = randomised; DB = double blund; wD = withdrawal or dropout; OPVS = Oxfoprd Pain validity Score; LOCF - last observation carried forward; ITT = intention to treat; N = number; LA = local anaesthetic; VAS = visual analogue scale; VRS = verbal rating scale; AE = adverse event; SPID = summed pain intensity difference; TOTPAR = total pain relief
