# Supplementary material for: Systematic review of dexketoprofen in acute and chronic pain
Source: BMC Clin Pharmacol. 2008 Oct 31;8:11. doi: 10.1186/1472-6904-8-11 (PMC2585070; doi:10.1186/1472-6904-8-11)
Supplement: Additional file 2 — Trials of oral and injected dexktoprofen in pain after surgery. The file contains information on each included study, with reference, quality score, design, treatments, main results, and comments. [file 1472-6904-8-11-S2.pdf]

Additional file 2: Trials of oral and injected dexktoprofen in pain after surgery

Reference	Methods	Details	Dosing regimen	Outcomes	Efficacy Results	Remedication, exclusions, and adverse events	Safety results	Quality score
**Oral administration**								
Berti et al. A prospective, randomised comparison of dexketoprofen, ketoprofen, or paracetamol for postoperative analgesia after outpatient knee arthroscopy. Minerva Anesteiologica 2000; 66:549-554	RCT, double oral dose, parallel groups, LA<br><br>Assessed during first 24 hrs and by telephone interview the following day - no further info??<br><br>Medication administered before nerve block placement and every 6/8 hrs thereafter	Knee arthroscopy<br><br>N= 45	Dexketoprofen 25mg BID<br>N= 15<br><br>Ketoprofen 50mg BID<br>N= 15<br><br>Paracetamol 500mg BID<br>N= 15	Pain Intensity (at rest)<br>100mm VAS<br><br>Pain Intensity (during motion)<br>100mm VAS<br><br>Pain<br>5-pt VRS<br><br>Quality of care	Mean VAS on movement significantly higher with paracetamol than other patients. Maximum pain moderate or severe in first 24 hours, 3 dexketoprofen, 6 ketoprofen, 5 paracetamol	No patients remedicated during their hospital stay, 2 patients remedicated following discharge, no info on missing data handled<br><br>No adverse events were reported	Dexketoprofen 25mg BID<br>No with >1 AE<br>AE withdrawals 0<br><br>Ketoprofen 50mg BID<br>No with >1 AE<br>AE withdrawals 0<br><br>Paracetamol 500mg BID<br>No with >1 AE<br>AE withdrawals 0	R 2<br>DB 0<br>WD 1<br><br>Total = 3<br><br>OPVS = 3/16
Ioham et al. Effect of perioperative administration of dexketoprofen on opioid requirements and inflammatory response following elective hip arthroplasty. Br J Anaesth 2002; 88: 520-526.	RCT, DB, 3 oral doses for three days, parallel groups, LA<br><br>Assessed at 24 and 18 hrs preoperatively, 6, 24, and 48 hrs postoperatively<br><br>Medication administered 25mg three times daily for 24hrs before and 48hrs after surgery. Following recovery all patients access to a PCA system with morphine	Hip arthroscopy<br><br>N= 30	Dexketoprofen 25mg TID<br>N= 15<br><br>Placebo<br>N= 15	Pain<br>VAS<br><br>Cumulative opioid consumption<br><br>Adverse events associated with opioid administration (nausea, respiratory depression, pruritus, sedation, urinary retention)<br>3/4-pt ordinal scales	Dexketoprofen 25mg TID<br>Cumulative morphine consumption 0.85mg<br>Time to first analgesia 1277 ± 1031 mins<br><br>Placebo<br>Cumulative morphine consumption 6mg<br>Time to first analgesia 642 ± 317 mins<br><br>Significantly lower morphine consumption with dexketoprofen than placebo	No adverse events attributable to dexketoprofen were reported, does not provide any information about un-related adverse events - may not have been collected	Withdrawals not reported	R 1<br>DB 1<br>WD 0<br><br>Total = 2<br><br>OPVS = 13/16
Zapata et al. Dexketoprofen vs tramadol: randomized double-blind trial in patients with postoperative pain. British J Clin Pharmacol 2000: 223 (abs 870).<br>Data from Harrison F: Double-blind randomised, parallel-group comparison of the safety and efficacy of oral dexketoprofen 25 mg with tramadol 50 mg in subjects with moderate to severe pain following orthopaedic surgery. Clinical Trial Report 2001.	RCT, DB, 8 oral doses, parallel groups<br><br>Assessed at baseline, 30 mins, 1, 2, 3, 4 and 5 hrs after the 1st dose; before and 2hrs after each following dose	Orthopaedic surgery<br><br>N= 187<br><br>17 centres in Germany, Belgium, and the Netherlands	Dexketoprofen trometamol 25mg<br>N= 93<br><br>Tramadol 50mg<br>N= 86	Pain Intensity<br>100mm VAS<br><br>Pain Intensity<br>4-pt VRS (0 - none, 1 - mild, 2- moderate, 3 - severe)<br><br>Pain Relief<br>5-pt VRS (0 - no relief, 1 - little, 2 - moderate, 3 - significant, 4 - complete)<br><br>Global efficacy (patient)<br>4-pt VRS<br><br>Global efficacy (investigator)<br>4-pt VRS<br><br>Time to onset<br><br>Half pain relieved (dichotomous scale)	Dexketoprofen trometamol 25mg<br>SPID6 13.9 ± 11.6<br>TOTPAR6 16.1 ± 5.4<br>Global good/excellent<br>Time to onset 2 ± 3<br>Time to remedication 11.6 ± 10.2<br><br>Tramadol 50mg<br>SPID6 7.8 ± 12<br>TOTPAR6 13.7 ± 6.2<br>Global good/excellent<br>Time to onset 3.6 ± 7.4<br>Time to remedication 6.2 ± 6.4<br><br>No significant differences between the two treatments	49 patients reported 69 adverse events, there were no serious adverse events and most were mild to moderate in severity	Withdrawals not reported	R 2<br>DB 1<br>WD 1<br><br>Total = 4<br><br>OPVS = 10/16

Vidal et al. Clinical trial to assess the analgesic efficacy and safety of LM-1158.TRIS (12.5 and 25 mg tid) versus ketoprofen (50 mg tid) and placebo after oral administration in patients with acute post-surgery pain. Clinical Trial report, 1999	RCT, DB, three oral doses, parallel groups, GA<br><br>Assessed at baseline, 15, 30 and 45 mins, and 1, 2, 3, 4, 5 and 6 hrs during the single dose phase, patients were assessed at the end of treatment (24 hrs) for the multiple dose phase<br><br>Medication administered once pain was rated as at least 'moderate', 2nd and 3rd doses were given 8 and 16 hrs later	Hallux valgus (bunion) surgery<br><br>N= 188<br><br>11 centres in Spain	Dexketoprofen trometamol 12.5mg TID<br>N= 47<br><br>Dexketoprofen trometamol 25mg TID<br>N= 47<br><br>Ketoprofen 50mg TID<br>N= 47<br><br>Placebo<br>N= 47	Pain Intensity<br>100mm VAS<br><br>Pain Intensity<br>4-pt VRS (0 - none, 1 - mild, 2- moderate, 3 - severe)<br><br>Pain Relief<br>5-pt VRS (0 - no relief, 1 - little, 2 - moderate, 3 - significant, 4 - complete)<br><br>Morphine usage<br><br>Global efficacy (patient)<br>4-pt VRS (excellent, good, mediocre, null)	Dexketoprofen trometamol 12.5mg TID<br>SPID6 77.8 ± 525.9<br>TOTPAR6 7.4 ± 17.1<br>Global good/excellent 27<br>Time to min pain intensity 24.7 ± 38.4<br>Time to remedication 138.7 ± 97.3<br>Morphine usage 9.2 ± 6.2<br><br>Dexketoprofen trometamol 25mg TID<br>SPID6 126.1 ± 485<br>TOTPAR6 7.4 ± 17.4<br>Global good/excellent 26<br>Time to min pain intensity 43.9 ± 71<br>Time to remedication 131.7 ± 92.2<br>Morphine usage 8.2 ± 5.1<br><br>Ketoprofen 50mg TID<br>SPID6 280.8 ± 567.8<br>TOTPAR6 2.7 ± 10.3<br>Global good/excellent 25<br>Time to min pain intensity 21.6 ± 48.1<br>Time to remedication 106 ± 50.4<br>Morphine usage 9.9 ± 6.7<br><br>Placebo<br>SPID6 347.3 ± 556.1<br>TOTPAR6 2.5 ± 7.7<br>Global good/excellent 17<br>Time to min pain intensity 12.6 ± 30.7<br>Time to remedication 100.5 ± 59.6<br>Morphine usage 12.7 ± 6.6<br><br>Single dose phase results showed no significant difference between groups	Patients remedicating during the 1st hr were withdrawn, patients remedicating after the 1st hr LOCF for pain intensity and pain relief set to 0<br><br>16 patients were excluded from efficacy analyses; 13 patients remedicated within the first hr, 2 patients had only mild pain, 1 patient had concurrent depression. All patients finished the single dose phase but 11 patients did not complete the multiple dose phase (5 due to AEs, 3 due to compliance issues, 2 treatment failures, 1 withdrew consent)<br><br>In total 112 patients reported 166 adverse events, there were significantly more patients experiencing adverse events; more placebo and dex 12.5mg reported at least one adverse event than dex 25mg	Dexketoprofen trometamol 12.5mg TID<br>No with >1 AE 33<br>All cause withdrawals 3<br>AE withdrawals 1<br><br>Dexketoprofen trometamol 25mg TID<br>No with >1 AE 20<br>All cause withdrawals 3<br>AE withdrawals 1<br><br>Ketoprofen 50mg TID<br>No with >1 AE 25<br>All cause withdrawals 0<br>AE withdrawals 0<br><br>Placebo<br>No with >1 AE 34<br>All cause withdrawals 5<br>AE withdrawals 2	R 2<br>DB 2<br>WD 1<br><br>Total = 5<br><br>OPVS = 13/16
Schreiber M. Double-blind, randomized, parallel-group comparison of the safety and efficacy of oral doses of dexketoprofen tromethamine salt (LM-1158.TRIS, 12.5 mg or 25 mg) with racemic ketoprofen (50 mg) and placebo in patients with moderate or severe pain following orthopaedic surgery. Clinical trial report, 1996.	RCT, DB, three oral doses for three days, parallel groups, LA or GA, 12 hr analgesic washout<br><br>Assessed at baseline, and 30 mins, and 1, 2, and 4 hrs after the 1st dose and baseline, 1 and 2 hrs after doses 2 to 9<br><br>Medication administered when pain described as moderate or severe within 4 hrs of surgery	Knee (meniscus or ligament reconstruction) or ankle surgery<br><br>N= 230	Dexketoprofen tromethamine 12.5mg TID<br>N=52<br><br>Dexketoprofen tromethamine 25mg TID<br>N=52<br><br>Ketoprofen 50mg TID<br>N=54<br><br>Placebo<br>N=55	Pain Intensity<br>100mm VAS<br><br>Pain Intensity<br>4-pt VRS (0 - absent, 1 - mild, 2- moderate, 3 - severe)<br><br>Pain Relief<br>5-pt VRS (0 - no relief, 1 - slight relief, 2 - moderate relief, 3 - considerable relief, 4 - complete relief)<br><br>Global efficacy (investigator)<br>4-pt VRS (not effective, mediocre, good, excellent)	Dexketoprofen tromethamine 12.5mg TID<br>4-hour TOTPAR 8.0<br><br>Dexketoprofen tromethamine 25mg TID<br>4-hour TOTPAR 9.03<br><br>Ketoprofen 50mg TID<br>4-hour TOTPAR 6.8<br><br>Placebo<br>4-hour TOTPAR 5.8<br><br>Both dexketoprofen doses significantly better than placebo, but not ketoprofen	Patients remedicating with 3g or more within 24 hrs or on 2 or more consecutive days were withdrawn<br><br>17 patients excluded for failing to adhere to GCP	Dexketoprofen tromethamine 12.5mg TID<br>No with >1 AE 2<br>All cause withdrawals 36<br>AE withdrawals 1<br><br>Dexketoprofen tromethamine 25mg TID<br>No with >1 AE 3<br>All cause withdrawals 35<br>AE withdrawals 2<br><br>Ketoprofen 50mg TID<br>No with >1 AE 0<br>All cause withdrawals 35<br>AE withdrawals 0<br><br>Placebo<br>No with >1 AE 3<br>All cause withdrawals 39<br>AE withdrawals 1	R 2<br>DB 2<br>WD 1<br><br>Total = 5<br><br>OPVS = 13/16

Perez et al. A multicentre clinical trial evaluating the analgesic efficacy and safety of dexketoprofen trometamol (25 mg tid) versus diclofenac (50 mg tid) for the treatment of pain subsequent to ambulatory surgery. Clinical trial report 2002	RCT, DB, three oral doses for three days, parallel groups, LA or GA, 12 hr analgesic washout<br><br>Assessed at baseline and after two hrs for each dose<br><br>Medication administered when the patient met all the discharge criteria or reported pain, mild pain included in the description of baseline demographics	Inguinal or rural herniorraphy<br><br>N= 173<br><br>7 centres in Spain	Dexketoprofen trometamol 25mg TID<br>N=83<br><br>Diclofenac 50mg TID<br>N=80	Pain Intensity<br>100mm VAS<br><br>Pain Relief<br>5-pt VRS (0 - no relief, 1 - slight relief, 2 - moderate relief, 3 - considerable relief, 4 - complete relief)<br><br>Global efficacy (patient)<br>4-pt VRS (excellent, good, mediocre, null)<br><br>Quality of sleep	Pain intensity over 8 doses declined in both grups, with significantly less pain with diclofenac 50mg TID than dexketoprofen 25mg TID at 4th and 8th doses, though no difference for pain relief		Dexketoprofen trometamol 25mg<br>No with >1 AE 20<br>AE withdrawals 0<br><br>Diclofenac 50mg<br>No with >1 AE 14<br>AE withdrawals 0	R 2<br>DB 2<br>WD 1<br><br>Total = 5<br><br>OPVS = 13/16
Schreiber M. Comparison of efficacy and tolerability of oral administration of 25 mg dexketoprofen (trometamol) vs 50 mg tramadol in patients with post-operative pain. Clinical trial report 1998	RCT, DB, parallel groups, 24 hr analgesic washout<br><br>Pan intensity 40/100 mm at baseline	Arthroscopy and other out-patient surgical procedures<br><br>14 centres in Germany	Dexketoprofen trometamol 25mg<br>N=93<br>38 included in ITT because of protocol violations<br><br>Tramadol 50mg<br>N=91<br>43 included in ITT because of protocol violations	Pain Intensity<br>100mm VAS<br>Rescue analgesics	No significant difference in pain intensity in a number of different analyses, nor in rescue medication used		Dexketoprofen trometamol 25mg<br>No with >1 AE<br>All cause withdrawals<br>AE withdrawals<br><br>Tramadol 50mg<br>No with >1 AE<br>All cause withdrawals<br>AE withdrawals	R 2<br>DB 2<br>WD 1<br><br>Total = 5<br><br>OPVS = 13/16
Latarjet J. A comparative study on safety and efficacy of dexketoprofen trometamol versus paracetamol codeine (Dafalgan Codeine) in the treatment of moderate to severe pain in the post-operative follow-up of hip-replacement surgery. Clinical trial report 1998.	R, DB, parallel groups, 3 daily doses over 3 days, GA,<br><br>Assessed at baseline, 4, 12, 20, 28, 36, 44, and 52 hrs<br><br>PCA morphine was also available	Hip replacement<br><br>25 anesthesiological teams in France	Dexketoprofen trometamol 25mg TID ± self-administered morphine<br>N= 100<br><br>Paracetamol 500mg ± codeine 22.5mg TID ± self-administered morphine<br>N= 100	Pain intensity<br>11-pt VRS<br><br>Morphine consumption<br><br>Global efficacy (patient)<br>4-pt VRS (good, relatively good, little satisfactory, poor)<br><br>Global efficacy (physician)<br>4-pt VRS (good, relatively good, little satisfactory, poor)<br><br>Sedation	No significant difference in morphine consumption or pain	A total of 76 patients reported 117 adverse events, there were no statistically significant differences between groups. 1 serious adverse event was reported.	Dexketoprofen trometamol 25mg TID<br>No with >1 AE 43<br>AE withdrawals 5<br><br>Paracetamol 500mg ± codeine 22.5mg TID<br>No with >1 AE 33<br>AE withdrawals 0	R 2<br>DB 2<br>WD 1<br><br>Total = 5<br><br>OPVS = 13/16
Tuncer et al. Postoperatif ağrida deksketoprofen kullanimi. Agri 2006 18:3	R, DB, parallel groups, oral dexletoprofen 25 mg 1 hour before and 8-16 hours after surgery,<br><br>Tramadol consumption from PCA	Abdominal hysterectomy<br><br>Turkey	Dexletoprofen 25 mg 1 hour before and 8-16 hours after surgery<br><br>Placebo<br><br>Group numbers not given, N=50 total	Tramadol consumption through PCA	Significantly less tramadol used by patients with dexketoprofen, and lower pain scores	No difference in adverse events		R 1<br>DB 0<br>WD 0<br><br>Total = 1<br><br>OPVS = 7/16

**Intramuscular and intravenous administration**								
Hanna et al. Comparative study of analgesic efficacy and morphine-sparing effect of intramuscular dexketoprofen with ketoprofen or placebo after major orthopaedic surgery. Br J Clin Pharmacol 2003; 55: 126-133.	RCT, DB, double IM dose, parallel groups, GA<br><br>Assessed immediately before morphine loading dose and second dose of study medication, at 2, 4, 6 and 9 hrs after the first dose, and 1, 9 and 12 hrs after the second dose<br><br>1st dose administered at the time of the first closing stitch (the end of surgery) with the 2nd dose administered 12 hrs later. Following recovery from anaesthesia, patients received titrated doses of 2-5mg of IV morphine and were connected to a PCA system with IV morphine	Orthopaedic surgery (hip or knee replacement)<br><br>N= 172<br><br>15 centres in the UK	Dexketoprofen trometamol 50mg IM BID ± morphine<br>N= 59<br><br>Ketoprofen 100mg IM BID ± morphine<br>N= 58<br><br>Placebo<br>N= 55	Total cumulative amount of morphine<br><br>Time to loading morphine dose<br><br>Time to first use of PCA morphine<br><br>Pain Intensity<br>10cm VAS<br><br>Pain Intensity<br>4-pt VRS (0 - none, 1 - mild, 2 - moderate, 3 - severe)<br><br>Quality of sleep<br>5-pt VRS (excellent, good, minor, discomfort, major discomfort, hardly slept at all)<br><br>Sedation scoring<br>4-pt VRS (0 - fully awake, 1 - mildly sedated, 2 - heavily sedated, 3 - fully awake)<br><br>Pain and/or discomfort at injection site<br><br>Blood loss from wound	Dexketoprofen trometamol 50mg IM BID<br>Morphine used 39.1mg<br>Time to loading dose 36mins<br>Time to PCA 78<br><br>Ketoprofen 100mg IM BID<br>Morphine used 41.3mg<br>Time to loading dose 38mins<br>Time to PCA 96<br><br>Placebo<br>Morphine used 64.8mg<br>Time to loading dose 26mins<br>Time to PCA 44<br><br>Significant reduction in morphine use for both active versus placebo. No difference in pain scores	LOCF used to input missing amounts of cumulative morphine usage<br><br>4 patients were not included as they lacked minimum valid measurement for morphine usage, 36 patients were withdrawn (lack of cooperation for 22 patients, adverse events for 4 patients, treatment failure for 3 patients and therapy success (refused 2nd dose as pain free at 12 hrs) for 6 patients, other reasons for 1 patient<br><br>139 patients reported 338 adverse events, there were no statistically significant differences between groups, 4 serious adverse events were reported (1 in the ketoprofen group and 3 with placebo)	Dexketoprofen trometamol 50mg IM BID<br>No with >1 AE NR<br>All cause withdrawals 10<br>AE withdrawals 0<br><br>Ketoprofen 100mg IM BID<br>No with >1 AE NR<br>All cause withdrawals 14<br>AE withdrawals 2<br><br>Placebo<br>No with >1 AE NR<br>All cause withdrawals 12<br>AE withdrawals 2	R 1<br>DB 1<br>WD 1<br><br>Total = 3<br><br>OPVS = 1o/16
Zippel H, Wagenitz A. Comparison of the efficacy and safety of intravenously administered dexketoprofen trometamol and ketoprofen in the management of pain after orthopaedic surgery. Clin Drug Invest 2006 26: 517-528.	RCT, DB, IV infusion 3 times per day over 2 days, parallel groups, GA, 12 hr analgesic washout<br><br>Assessed at baseline, 15 and 30 mins, and 1, 2, 4, 6, 8, 16, 24, 32 and 48 hrs<br><br>Medication administered when pain intensity was >40mm on a 10mm VAS and within 12 hrs of recovery from GA at 0, 8, 16, 24, 32 and 40 hrs	Orthopaedic surgery (hip or knee replacement)<br><br>N= 252<br><br>11 centres in Belgium, France, Germany and South Africa	Dexketoprofen trometamol 50mg IV TID<br>N= 125<br><br>Ketoprofen 100mg IV TID<br>N= 127	Pain Intensity<br>100mm VAS	Dexketoprofen trometamol 50mg IV TID<br>SPID8 280.4 ± 17.1<br>Time to max PID 284.7 ± 165.9<br>Time to remedication 3.49 ± 5.96<br>No remedicating 91<br><br>Ketoprofen 100mg IV TID<br>SPID8 302.2 ± 17.1<br>Time to max PID 308.5 ± 151.5<br>Time to remedication 4.28 ± 8.1<br>No remedicating 95<br><br>No significant differences between the two treatment	Remedication permitted, patients remedicating within the first 30 mins were withdrawn. Missing VAS scores due to patient sleeping were inputted as 0, LOCF used for patients withdrawing after first 30 min due to adverse events or missing more than one VAS score<br><br>5 patients were excluded from the ITT analysis, all due to missing baseline or post-baseline measurements<br><br>132 patients reported 223 adverse events, there were fewer events in the dexketoprofen group (49% v 56%), 6 patients experienced serious adverse events (3 per group), 3 patients withdrew as a result of adverse events	Dexketoprofen trometamol 50mg IV TID<br>No with >1 AE 61<br>AE withdrawals 1<br><br>Ketoprofen 100mg IV TID<br>No with >1 AE 71<br>AE withdrawals 2	R 2<br>DB 2<br>WD 1<br><br>Total = 5<br><br>OPVS = 13/16

Peat S. Double blind, randomised, parallel group study of the safety, efficacy and influence on morphine usage of intravenous dexketoprofen trometamol (50 mg) in comparison to intravenous tramadol ((100 mg) or placebo in the relief of pain following orthopaedic surgery. Clinica trial report 2000	RCT, DB, DD, double dose, parallel groups, GA, 6hr analgesic washout<br><br>Assessed at 30 mins and 1, 2, 3, 4, 6, 10 and 12 hrs<br><br>Medication administered approx 30 mins before anticipated waking time and the second dose 6hrs later. All patients were connected to a PCA system with IV morphine and received a loading dose if required	Orthopaedic surgery (hip or knee replacement)<br><br>N= 215<br><br>18 centres in Belgium, the Netherlands, South Africa and the UK	Dexketoprofen 50mg IV BID<br>N= 73<br><br>Tramadol 100mg IV BID<br>N= 73<br><br>Placebo<br>N= 69	Pain Intensity<br>100mm VAS<br><br>Hourly rate of morphine usage<br><br>Time to first PCA demand<br><br>Sedation<br>4-pt ordinal scale (fully awake, mildly sedated, heavily sedated asleep)	Dexketoprofen 50mg IV BID<br>SPID6 175.5<br>SPID12 259.8<br>Mean morphine consumption 20.1 ±12<br>Hourly morphine consumption 1.7 ± 1<br>Time to first PCA use 72.6 ± 56.2<br>No of PCA demands 3.68 ± 4.5<br><br>Tramadol 100mg IV BID<br>SPID6 204.6<br>SPID12 306.1<br>Mean morphine consumption 19.6 ± 10.2<br>Hourly morphine consumption 1.6 ± 0.8<br>Time to first PCA use 68.1 ± 44.9<br>No of PCA demands 3.64 ± 4.54<br><br>Placebo<br>SPID6 220.7<br>SPID12 367.5<br>Mean morphine consumption 26.8 ± 12.2<br>Hourly morphine consumption 2.2 ± 1<br>Time to first PCA use 57.6 ± 28.9<br>No of PCA demands 4.42 ± 3.8<br><br>Pain scores and morphine requirements similar in both active groups, and both significantly better than placebo	For patients withdrawing due to adverse events or therapeutic efficacy LOCF used, for patients withdrawing due to lack of efficacy, the maximum VAS score from baseline to the last measurement was carried forward<br><br>7 patients were excluded as the PCA morphine was not set up and 4 patients did not receive two doses of study medication<br><br>104 patients reported 179 adverse events, 4 patients experienced serious adverse events (1 dexketoprofen patient and 3 tramadol patients) - 2 of which died for reasons unrelated to the study drugs	Dexketoprofen 50mg IV BID<br>No with >1 AE 37<br>All cause withdrawals 2<br>AE withdrawals 1<br><br>Tramadol 100mg IV BID<br>No with >1 AE 35<br>All cause withdrawals 4<br>AE withdrawals 1<br><br>Placebo<br>No with >1 AE 32<br>All cause withdrawals 2<br>AE withdrawals 1	R 2<br>DB 2<br>WD 1<br><br>Total = 5<br><br>OPVS = 13/16

Puig et al. Multicentre clinical trial to assess the efficacy and safety of dexketoprofen trometamol (25 mg and 50 mg bid) versus diclofenac (75 mg bid) by the intramuscular route in the treatment of postoperative pain. Clinical trial report 2000	RCT, DB, double IM dose, parallel groups<br><br>Assessed at baseline, 15, 30 and 45 mins, and 1, 1.5, 2, 3, 4, 5, 6, and 8 hrs<br><br>Medication administered when pain >30mm within 12 hrs of surgery	Abdominal gynaecological (non laparoscopic) surgery<br><br>N= 340<br><br>22 centres in Spain, Denmark and Sweden	Dexketoprofen trometamol 25mg IM BID<br>N= 74<br><br>Dexketoprofen trometamol 50mg IM BID<br>N= 71<br><br>Diclofenac 75mg IM BID<br>N= 68<br><br>Placebo<br>N= 71	Pain Intensity<br>100mm VAS<br><br>Pain relief<br>5-pt VRS (0 - no pain to 4 - complete relief)<br><br>Morphine consumption<br><br>Overall assessment of efficacy<br><br>Quality of sleep<br><br>Sedation<br>4-pt VRS (0 - awake to 3 - asleep)	Dexketoprofen trometamol 25mg IM BID<br>SPID6 3.3 ± 4.1<br>SPID8 4.1 ± 5.3<br>TOTPAR6 9.1 ± 7.3<br>TOTPAR8 10.8 ± 9.6<br>Time to max PID 60 (30 - 120)<br>No remedicating (1st dose) 51<br>Morphine consumption (1st dose) 5.5 ± 7.8<br><br>Dexketoprofen trometamol 50mg IM BID<br>SPID6 5± 4.7<br>SPID8 6.2 ± 6<br>TOTPAR6 12.7 ± 8.2<br>TOTPAR8 16 ± 11.1<br>Time to max PID 60 (30 - 120)<br>No remedicating (1st dose) 32<br>Morphine consumption (1st dose) 3.3 ± 6.5<br><br>Diclofenac 75mg IM BID<br>SPID6 4.3 ± 4.5<br>SPID8 5.8 ± 6.1<br>TOTPAR6 11.4 ± 8.4<br>TOTPAR8 14.5 ± 11.4<br>Time to max PID 60 (30 - 240)<br>No remedicating (1st dose) 35<br>PlaceboSPID6 1.4 ± 4.2<br>SPID8 1.6 ± 5.6<br>TOTPAR6 7.9 ± 7<br>TOTPAR8 9.5 ± 9.2<br>Time to max PID 30 (15 - 90)<br>No remedicating (1st dose) 50<br>Morphine consumption (1st dose) 5.2 ± 5.6<br><br>All active groups were produced significantly more analgesia than placebo, with dexketoprofen having better pain scores than diclofenac at times between 3 and 8 hours. Less morphine needed with dexketoprofen 50 mg and diclofenac than placebo and dexketoprofen 25 mg	39 patients were excluded from efficacy analyses due to being included prior to a protocol amendment<br><br>A total of 310 adverse events were reported by 201 patients, most were mild to moderate in intensity (20 severe cases were reported), there were no statistically significant differences between groups. 11 serious adverse events were reported in 9 patients (1 with placebo, 1 with diclofenac, 3 with dex 25mg, and 4 with dex 50mg	Dexketoprofen trometamol 25mg IM BID<br>No with >1 AE 50<br>All cause withdrawals 10<br>AE withdrawals 0<br><br>Dexketoprofen trometamol 50mg IM BID<br>No with >1 AE 52<br>All cause withdrawals 4<br>AE withdrawals 0<br><br>Diclofenac 75mg IM BID<br>No with >1 AE 45<br>All cause withdrawals 5<br>AE withdrawals 1<br><br>Placebo<br>No with >1 AE 54<br>All cause withdrawals 7<br>AE withdrawals 1	R 2<br>DB 2<br>WD 1<br><br>Total = 5<br><br>OPVS = 13/16

Abbreviations: RCT = randomised controlled trial; R = randomised; DB = double blund; wD = withdrawal or dropout; OPVS = Oxfoprd Pain validity Score; LOCF - last observation carried forward; ITT = intention to treat; N = number; LA = local anaesthetic; VAS = visual analogue scale; VRS = verbal rating scale; AE = adverse event; SPID = summed pain intensity difference; TOTPAR = total pain relief
