# Supplementary material for: Systematic review of dexketoprofen in acute and chronic pain
Source: BMC Clin Pharmacol. 2008 Oct 31;8:11. doi: 10.1186/1472-6904-8-11 (PMC2585070; doi:10.1186/1472-6904-8-11)
Supplement: Additional file 3 — Trials of injected dexktoprofen in pain of renal colic. The file contains information on each included study, with reference, quality score, design, treatments, main results, and comments. [file 1472-6904-8-11-S3.pdf]

Additional file 3: Trials of injected dexktoprofen in pain of renal colic

Reference	Methods	Details	Dosing regimen	Outcomes	Efficacy results	Remedication, exclusions, and adverse events	Safety results	Quality score
Sanchez Carpena et al. Comparison of dexketoprofen trometamol and dipyrone in the treatment of renal colic. Clin Drug Invest 2003; 23: 139-152.	R, DB, parallel group, single IM dose, 2 hr analgesic washout<br><br>Assessed at baseline, 10, 20, 30 and 45 mins, and 1, 2, 4 and 6 hrs<br><br>Patients had at least moderate pain intensity due to suspected renal colic immediately before study drug administration	Renal colic<br><br>N= 333<br><br>18 centres in Spain, Sweden, and Finland	Dexketoprofen trometamol 25mg IM<br>N= 112<br><br>Dexketoprofen trometamol 50mg IM<br>N= 113<br><br>Dipyrone 2g IM<br>N= 108	Pain intensity<br>100mm VAS<br><br>Pain intensity<br>4-pt VRS (0 - none, 1 - mild, 2 - moderate, 3 - severe)<br><br>Pain relief<br>5-pt VRS (0 - no relief, 1 - slight, 2 - moderate, 3 - significant, 4 - complete)<br><br>Patient global<br>4-pt VRS (0 - null, 1 - poor, 2 - good, 3 - excellent)	Dexketoprofen trometamol 25mg IM<br>SPID6 8.3 + 6<br>TOTPAR6 14.4 + 8.2<br>Global good/excellent 78.2%<br>Time to max PID 60 (45 - 180)<br>Time to remedication 127.5 (72.5 - 195)<br>No remedicating 40<br><br>Dexketoprofen trometamol 50mg IM<br>SPID6 8.7 + 6.4<br>TOTPAR6 15.1 + 8.5<br>Global good/excellent 72.2%<br>Time to max PID 60 (30 - 120)<br>Time to remedication 120 (65 - 154)<br>No remedicating 33<br><br>Dipyrone 2g IM<br>SPID6 8.1 + 6<br>TOTPAR6 14.2 + 8.2<br>Global good/excellent 74.2<br>Time to max PID 120 (45 - 120)<br>Time to remedication 160 (60 - 240)<br>No remedicating 35<br><br>No significant difference between the three treatments	Patients withdrawing due to adverse events, lack of efficacy or remedication after the first time point were assigned maximum pain intensity and pain relief set to 0, LOCF used for other reasons for withdrawal<br><br>8 patients treated with dex 25mg were excluded from efficacy analyses (6 colic not confirmed, 2 insufficient pain, 1 rescue within 30mins), 12 from dex 50mg (9 colic not confirmed, 2 insufficient pain, 1 rescue within 30mins), and 11 patients from the dipyrone group (8 colic not confirmed, 2 rescue within 30mins, and 1 lack of evaluations)<br><br>A total of 76 patients experienced 103 adverse events, most events were of mild to moderate intensity though 9 cases were classed as severe. 20 serious adverse events were reported (8 with dex 25mg, and 6 in each other group)	Dexketoprofen trometamol 25mg IM<br>No with >1 AE 24<br>All cause withdrawals 10<br>AE withdrawals 0<br><br>Dexketoprofen trometamol 50mg IM<br>No with >1 AE 24<br>All cause withdrawals 14<br>AE withdrawals 1<br><br>Dipyrone 2g IM<br>No with >1 AE 28<br>All cause withdrawals 14<br>AE withdrawals 3	R 2<br>DB 1<br>WD 1<br><br>Total = 4<br><br>OPVS = 10/16

Sanchez Carpena et al. Comparison of intravenous dexketoprofen and dipyrone in acute renal colic. Eur J Clin Pharmacol 2007; 63: 751-760.	R, DB, DD, parallel group, single IV dose, 2 hr analgesic washout<br><br>Assessed at baseline, 10, 20, 30 and 45 mins, and 1, 2, 4 and 6 hrs<br><br>Patients had at least moderate pain intensity due to suspected renal colic immediately before study drug administration	Renal colic<br><br>N= 308<br><br>17 centres in Spain	Dexketoprofen 25mg IV<br>N= 101<br><br>Dexketoprofen 50mg IV<br>N= 104<br><br>Dipyrone 2g IV bolus<br>N= 103	Pain intensity<br>100mm VAS<br><br>Pain intensity<br>4-pt VRS (0 - none, 1 - mild, 2 - moderate, 3 - severe)<br><br>Pain relief<br>5-pt VRS (0 - no relief, 1 - slight, 2 - moderate, 3 - significant, 4 - complete)	Dexketoprofen 25mg IV<br>SPID6 8.1 + 7<br>TOTPAR6 13.5 + 8.6<br>Global good/excellent NR<br>Time to max PID 45 (30 - 120)<br>Time to remedication 127.5 (70 - 190)<br>No remedicating 34 (33.7)<br><br>Dexketoprofen 50mg IV<br>SPID6 9.8 + 6.2<br>TOTPAR6 15.3 + 8.6<br>Global good/excellent NR<br>Time to max PID 45 (30 - 240)<br>Time to remedication 115 (65 - 175)<br>No remedicating 26 (25)<br><br>Dipyrone 2g IV bolus<br>SPID6 10.3 + 5.4<br>TOTPAR6 15.5 + 7.9<br>Global good/excellent NR<br>Time to max PID 60 (30 - 120)<br>Time to remedication 150 (82.5 - 360)<br>No remedicating 16 (15.5)	Patients withdrawing due to adverse events, lack of efficacy or remedication after the first time point were assigned maximum pain intensity and pain relief set to 0, LOCF used for other reasons for withdrawal<br><br>No patients were excluded from the ITT analyses<br><br>A total of 152 patients experienced 220 adverse events, most were mild to moderate in severity. 13 serious events were reported (5 in each dexketoprofen group, 3 with dipyrone), most were recurrence of renal pain that led to hospitalisation. 5 patients were withdrawn due to adverse events (2 in the dexketoprofen 25mg group, 1 in the 50mg group, and 2 in the dipyrone group).	Dexketoprofen 25mg IV<br>No with >1 AE 17<br>All cause withdrawals 7<br>AE withdrawals 2<br><br>Dexketoprofen 50mg IV<br>No with >1 AE 20<br>All cause withdrawals 9<br>AE withdrawals 1<br><br>Dipyrone 2g IV bolus<br>No with >1 AE 10<br>All cause withdrawals 15<br>AE withdrawals 2	R 2<br>DB 2<br>WD 1<br><br>Total = 5<br><br>OPVS = 13/16
Debre B. A double-blind (observer blind), randomized trial comparing the analgesic efficacy and safety of dexketoprofen trometamol (50 mg) with ketoprofen (100mg) given as an intravenous infusion, in patients with renal or urethral colic. Clinical trial report 2000	R, DB, parallel group, single IV dose, patients treated with NSAIDs/opiates within 24 hrs, long half-life NSAIDs within 72hrs, or corticosteroids within 1 wk, were excluded<br><br>Assessed at 15, 30 and 45 mins, and 1, 2, 3, 4 and 6 hrs<br><br>Patients had at least >50mm pain intensity due to suspected renal colic immediately before study drug administration	Renal or urethral colic<br><br>N= 198<br><br>25 centres in France and Belgium	Dexketoprofen trometamol 50mg IV<br>N= 96<br><br>Ketoprofen 100mg IV<br>N= 101	Pain intensity<br>100mm VAS	Dexketoprofen trometamol 50mg IV<br>SPID6 358.7 + 10.98<br>SPID6 (no criterion) 114.4 + 44.15<br>Time to max PID 2.42 + 2.09<br>Time to remedication 3.3hrs approx<br>No remedicating 17%<br><br>Ketoprofen 100mg IV<br>SPID6 368.7 + 10.59<br>SPID6 (no criterion) 112.7 + 42.31<br>Time to max PID 2.12 + 1.82<br>Time to remedication 3.3hrs approx<br>No remedicating 17%<br><br>No significant difference between the treatments	LOCF used to input missing data for patients withdrawing for reasons other than lack of efficacy<br><br>5 patients were excluded from ITT analyses; 1 patient dropped out prior to randomisation, 1 patient did not receive medication, data was missing for 3 patients<br><br>A total of 40 patients reported 57 adverse events, most were of mild to moderate intensity, although 6 per group were classed as severe, 3 patients experienced 4 serious adverse events (2 in the ketoprofen group, 1 in the dex group)	Dexketoprofen trometamol 50mg IV<br>No with >1 AE 17<br>All cause withdrawals 4<br>AE withdrawals 0<br><br>Ketoprofen 100mg IV<br>No with >1 AE 23<br>All cause withdrawals 3<br>AE withdrawals 1	R 2<br>DB 1<br>WD 1<br><br>Total = 4<br><br>OPVS = 10/16

Abbreviations: RCT = randomised controlled trial; R = randomised; DB = double blund; wD = withdrawal or dropout; OPVS = Oxfoprd Pain validity Score; LOCF - last observation carried forward; ITT = intention to treat; N = number; LA = local anaesthetic; VAS = visual analogue scale; VRS = verbal rating scale; AE = adverse event; SPID = summed pain intensity difference; TOTPAR = total pain relief
