# Supplementary material for: Systematic review of dexketoprofen in acute and chronic pain
Source: BMC Clin Pharmacol. 2008 Oct 31;8:11. doi: 10.1186/1472-6904-8-11 (PMC2585070; doi:10.1186/1472-6904-8-11)
Supplement: Additional file 4 — Trials of intramuscular and oral dexktoprofen in acute back pain. The file contains information on each included study, with reference, quality score, design, treatments, main results, and comments. [file 1472-6904-8-11-S4.pdf]

Additional file 4: Trials of intramuscular and oral dexktoprofen in acute back pain

Reference	Methods	Details	Dosing regimen	Outcome measures	Efficacy results	Remedication, exclusions, and adverse events	Safety results	Quality score
**Intramuscular**								
Zippel H, Wagenitz A. A multicentre, randomised, double-blind study comparing the efficacy and tolerability of intramuscular dexketoprofen versus diclofenac in the symptomatic treatment of acute low back pain. Clin Drug Investig 2007; 27:533-543.	RCT, DB, two IM doses over 2 days, parallel groups, 6 hr analgesic washout<br><br>Assessed at (1st dose) baseline, 30, 60, and 90 mins, 2, 4, and 6 hrs; (2nd dose) baseline, 1, 2, 4, 6, and 8 hrs; (3rd dose) baseline, 1, 2, 4 and 6 hrs; (4th dose) baseline and 2 hrs<br><br>Acute back pain of no more than 1 wk duration and moderate to severe intensity (≥50mm VAS)	Low back pain of less than 1 week duration<br><br>N= 370<br><br>21 centres in Belgium, Germany, and Poland	Dexketoprofen 50mg IM BID<br>N= 183<br><br>Diclofenac 75mg IM BID<br>N= 187	Pain intensity<br>100mm VAS<br><br>Roland disability questionaire	Dexketoprofen 50mg<br>SPID6 114.5 ± 104.66<br>Multiple-dose phase<br>SPIDlast 295.6 ± 206.7<br>Time to peak 3.6 ± 2.1<br>No remedicating 72<br><br>Diclofenac 75mg<br>SPID6 112.7 ± 105.71<br>Multiple-dose phase<br>SPIDlast 284.2 ± 20<br>Time to peak 3.7 ± 2.0<br>No remedicating 62<br><br>There were no significant differences in short or longer term	Remedication permitted<br><br>All randomised patients included in the ITT analysis; 20 patients withdrawn (10 per group)<br><br>A total of 108 patients reported 236 adverse events, there were no significant differences between groups, and the majority of events were mild or moderate in severity	Dexketoprofen 50mg<br>No with >1 AE 50<br>All cause withdrawals 10<br>AE withdrawals 4<br><br>Diclofenac 75mg<br>No with >1 AE 58<br>All cause withdrawals 10<br>AE withdrawals 2	R 2<br>DB 2<br>WD 1<br><br>Total = 5<br><br>OPVS = 13/16
**Oral**								
Kubler U. Comparative clinical trial of the efficacy and tolerability of 25 mg dexketoprofen tid versus 50 mg tramadol tid in patients with acute lumbago of at least moderate severity. Clinical trial report 1999. Also published as: Metscher et al. [Dexketoprofen-trometamol and tramadol in acute lumbago]. Fortschr Med Orig 2001, 118(4):147-151.	RCT, DB, 3 daily doses over 7 days, parallel group,<br><br>Assessed at baseline, day 4, and day 8<br><br>Medication administered to patients with untreated back pain of at least moderate intensity (50mm) of no more than 48 hrs duration	Acute lumbago of less than 2 days duration<br><br>N= 193<br><br>25 centres in Germany	Dexketoprofen 25mg TID<br>N= 97<br><br>Tramadol 50mg TID<br>N= 95	Pain on movement<br>100mm VAS<br><br>Pain at rest<br>100mm VAS<br><br>Nocturnal pain<br>100mm VAS<br><br>Schroberg's test of function for lumbar spine<br><br>Global improvement - patient<br>7-pt VRS (very much improved, much improved, only slightly improved, unchanged, slightly worse, very much worse)<br><br>Global improvement - physician<br>7-pt VRS (very much improved, much improved, only slightly improved, unchanged, slightly worse, very much worse)<br><br>Global evaluation of efficacy - patient<br>4-pt VRS (very good, good, moderate, no effect)<br><br>Global evaluation of efficacy - physician<br>4-pt VRS (very good, good, moderate, no effect)	Dexketoprofen 25mg TID<br>Median onset 60mins<br>Median duration of effect 155mins<br>Pain on movement 78.9 to 17.6mm<br>Pain at rest 73.7 to 15.9mm<br>Nocturnal pain 44.7 to 2.5mm<br>Restriction of movement AM 77.5 to 17mm<br>Global improvement (patient) improved/very much improved 82<br>Global efficacy (patient) good/very good 81<br>Global improvement (physician) improved/very much improved 84<br>Global efficacy (physician) good/very good 85<br><br>Tramadol 50mgTID<br>Median onset 65mins<br>Median duration of effect 165mins<br>Pain on movement 79.2 to 24.1mm<br>Pain at rest 72.7 to 19.9mm<br>Nocturnal pain 41.4 to 7.1mm<br>Restriction of movement AM 73.2 to 22.1mm<br>Global improvement (patient) improved/very much improved 73<br>Global efficacy (patient) good/very good 70<br>Global improvement (physician) improved/very much improved 66<br>Global efficacy (physician) good/very good 62<br><br>No major differences between the two treatments, though some outcomes and adverse events better for dexketoprofen	Remedication permitted. Data for patient who withdrew before day 4 was excluded, LOCF used for patients withdrawing after day 4<br><br>1 patient was lost to follow up and excluded from analyses, 5 patients withdrew before day 4<br><br>A total of 41 patients reported 56 adverse events, significantly more adverse events occurred in the tramadol treatment group (15 v 26, p=0.04). No serious adverse events were reported, 6 patients withdrew as a result of adverse events.	Dexketoprofen trometamol 25mg TID<br>No with >1 AE 15<br>All cause withdrawals 16<br>AE withdrawals 2<br><br>Tramadol 50mg TID<br>No with >1 AE 26<br>All cause withdrawals 13<br>AE withdrawals 4	R 2<br>DB 2<br>WD 1<br><br>Total = 5<br><br>OPVS = 13/16

Granados et al. Clinical trial to assess the efficacy and safety of LM-1158.tris (25 mg tid) versus diclofenac (50 mg) in the symptomatic treatment of patients with acute lumbar pain. Clinical trial report 1999	RCT, DB, three daily doses over 2 wks, parallel groups, 6 hr NSAID washout<br><br>Pain intensity assessed daily during the 1st wk, all other assessments at the end of wk 1 and wk 2<br><br>Medication administered to patients with back pain of at least moderate intensity (30mm) and history of previous episode within 6 months	Acute lumbar pain with no previous episodes within 6 months<br><br>N= 63<br><br>4/6 centres in Spain	Dexketoprofen trometamol 25mg TID N=32<br><br>Diclofenac 50mg TID N=31	Pain intensity<br>100mm VAS<br><br>Pain intensity<br>4-pt VRS (0 - no pain, 1 - mild, 2 - moderate, 3 - severe pain)<br><br>Schroberg's of function for lumbar spine<br><br>Global evolution of lumbar pain - patient<br>5-pt VRS (0 - much worse to 4 - much better OR 1 - much worse to 5 - much better)<br><br>Global evolution of lumbar pain - physician<br>5-pt VRS (0 - much worse to 4 - much better OR 1 - much worse to 5 - much better)	Dexketoprofen trometamol 25mg TID<br>Pain intensity (VAS) wk1 66.3, wk2 25.8, wk3 14mm<br>Global evolution of lumbar pain (patient) little better/much better 88.9<br>Global evolution of lumbar pain (physician) little better/much better 96.3%<br><br>Diclofenac 50mg TID<br>Pain intensity (VAS) wk1 54.1, wk2 25.9, wk3 14.7mm<br>Global evolution of lumbar pain (patient) little better/much better 96.4%<br>Global evolution of lumbar pain (physician) little better/much better 100%<br><br>No significant difference between the two treatments	Remedication permitted<br><br>8 patients were excluded from analyses as lost to follow up within the 1st wk<br><br>In total 10 patients reported 12 adverse events, there were no significant differences between groups and all events were mild to moderate in intensity. No serious adverse events were reported	Dexketoprofen trometamol 25mg TID<br>No with >1 AE 3<br>All cause withdrawals 11<br>AE withdrawals 1<br><br>Diclofenac 50mg TID<br>No with >1 AE 7<br>All cause withdrawals 10<br>AE withdrawals 2	R 2<br>DB 2<br>WD 1<br><br>Total = 5<br><br>OPVS = 12/16
Castiaux. Comparative, multicentre, randomised, double-blind, parallel-group trial on the efficacy and tolerability of dexketoprofen trometamol 25 mg tid, versus tramadol 50 mg tid in a 7-day treatment of acute low back pain. Clinical trial report 1999	RCT, DB, 3 daily doses over 7 days, parallel group, 6 hr analgesic washout<br><br>Pain intensity assessed at 15 and 30 mins after morning dose and at baseline and day 7, all other assessments made at baseline and day 7<br><br>Medication administered to patients with untreated back pain of at least moderate intensity (50mm) of no more than 4 days duration	Low back pain within last 4 days<br><br>N= 310<br><br>42 centres in Belgium and Germany	Dexketoprofen trometamol 25mg TID N= 155<br><br>Tramadol 50mg TID N= 152	Pain intensity<br>100mm VAS<br><br>Pain at rest - physician<br>4-pt VRS (0 to 3)<br><br>Pain on palpitation - physician<br>4-pt VRS (0 to 3)<br><br>Pain on movement - physician<br>4-pt VRS (0 to 3)<br><br>Schubert index<br><br>Doodads functional index<br><br>Night pain<br><br>Mobility<br>4-pt VRS (0 to 3)<br><br>Spinal contracture<br>4-pt VRS (0 to 3)<br><br>Global efficacy - patient<br>4-pt VRS (0 to 3)<br><br>Global efficacy - physician<br>4-pt VRS (0 to 3)	Dexketoprofen trometamol 25mg TID<br>Pain after treatment - patient 75.8 to 21.7mm<br>Change in pain at rest (physician) none/mild 146<br>Change in pain on palpitation (physician) none/mild 136<br>Change in pain on movement (physician) none/mild 124<br>Global efficacy (patient) good/very good 112<br>Global efficacy (physician) good/very good 112<br><br>Tramadol 50mg TID<br>Pain after treatment - patient 75.7 to 26.6mm<br>Change in pain at rest (physician) none/mild 134<br>Change in pain on palpitation (physician) none/mild 120<br>Change in pain on movement (physician) none/mild 117<br>Global efficacy (patient) good/very good 97<br>Global efficacy (physician) good/very good 100<br><br>No significant difference between the two treatments	3 patients were excluded from the ITT analyses, 2 were lost to follow-up and no information for post-treatment evaluation was available for 1 patient<br><br>In total 95 patients reported 179 adverse events, there were significantly fewer adverse events with dexketoprofen (23%) than tramadol (39%)	Dexketoprofen 25mg TID<br>No with >1 AE 36<br>All cause withdrawals 23<br>AE withdrawals 2<br><br>Tramadol 50mg TID<br>No with >1 AE 59<br>All cause withdrawals 42<br>AE withdrawals 20	R 2<br>DB 2<br>WD 1<br><br>Total = 5<br><br>OPVS = 13/16

Bourgeois P. Multicentre, comparative, double blind study of dexketoprofen trometamol 25 mg versus Di-antalvic in the treatment of acute low back pain. Clinical trial report 1999	RCT, DB, 3 daily doses over 3 days, parallel groups,<br><br>Patient assessed pain intensity at baseline, 1, and 4 hrs after administration, at night on day 1, and prior to medication in the morning and evening of day 2 and 3. Other assessment done on day 1 and day 4.<br><br>Medication administered to patients with back pain less than or equal to 50mm and of no more than 5 days duration	Low back pain within last 5 days<br><br>N=336<br><br>67 centres in France	Dexketoprofen trometamol 25mg TID<br>N= 168<br><br>Dextropropoxyphene 30mg + paracetamol 400mg BID<br>N= 167	Pain intensity<br>100mm VAS<br><br>Lumbar pain - physician<br>4-pt VRS (0 - absent, 1 - mild, 2 - moderate, 3 - severe)<br><br>Global efficacy - patient<br>4-pt VRS (1 - excellent, 2 - good, 3 - moderate, 4 - poor)<br><br>Global efficacy - physician<br>4-pt VRS (1 - excellent, 2 - good, 3 - moderate, 4 - poor)<br><br>Eiffel function index<br><br>Lumbar contracture - physician<br>4-pt VRS (0 - absent, 1 - mild, 2 - moderate, 3 - severe)	Dexketoprofen trometamol 25mg TID<br>Global efficacy (patient) good/excellent 78%<br>Global efficacy (physician) good/excellent 82%<br><br>Dextropropoxyphene 30mg ± paracetamol 400mg BID<br>Global efficacy (patient) good/excellent 63%<br>Global efficacy (physician) good/excellent<br><br>No significant difference in pain between the two treatments	1 patient was excluded from the ITT analyses for failing to attend post-treatment follow-up	Dexketoprofen trometamol 25mg TID<br>No with >1 AE 21<br>All cause withdrawals<br>AE withdrawals 6<br><br>Dextropropoxyphene 30mg ± paracetamol 400mg BID<br>No with >1 AE 18<br>All cause withdrawals<br>AE withdrawals 2	R 2<br>DB 2<br>WD 1<br><br>Total = 5<br><br>OPVS = 13/16

Abbreviations: RCT = randomised controlled trial; R = randomised; DB = double blund; wD = withdrawal or dropout; OPVS = Oxfoprd Pain validity Score; LOCF - last observation carried forward; ITT = intention to treat; N = number; LA = local anaesthetic; VAS = visual analogue scale; VRS = verbal rating scale; AE = adverse event; SPID = summed pain intensity difference; TOTPAR = total pain relief
