# Supplementary material for: Systematic review of dexketoprofen in acute and chronic pain
Source: BMC Clin Pharmacol. 2008 Oct 31;8:11. doi: 10.1186/1472-6904-8-11 (PMC2585070; doi:10.1186/1472-6904-8-11)
Supplement: Additional file 5 — Trials of oral dexktoprofen in gynaecological and other acute painful conditions. The file contains information on each included study, with reference, quality score, design, treatments, main results, and comments. [file 1472-6904-8-11-S5.pdf]

Additional file 5: Trials of oral dexktoprofen in gynaecological and other acute painful conditions

Reference	Methods	Details	Dosing regimen	Outcomes	Efficacy results	Remedication, exclusions, and adverse events	Safety results	Quality score
**Gynaecological**								
Ezcurdia et al. Comparison of the efficacy and tolerability of dexketoprofen and ketoprofen in the treatment of primary dysmenorrhoea. J Clin Pharmacol 1998; 38(12 Suppl): 65S-73S.	R, DB, oral doses, crossover, 12 hr analgesic washout<br><br>During the single dose phase, patients were assessed at baseline, 0.5, 1, 2, 3, 4 and 6 hrs. During the repeated dose phase patients returned to study centre at the end of each cycle to complete evaluations and for allocation of medication for next cycle<br><br>All women had a history of dysmenorrhoea of at least 4 months duration and moderate to severe pain necessitating the use of analgesics in at least 75% of cycles, patients were instructed to start treatment when menstrual pain became moderate to severe	Primary dysmenorrhoea<br><br>N= 52<br><br>5 centres in Spain	Each patient received a different treatment for each of four consecutive menstrual cycles<br><br>Dexketoprofen 12.5mg<br><br>Dexketoprofen 25mg<br><br>Ketoprofen (racemic) 50mg<br><br>Placebo	Pain intensity<br>100mm VAS<br><br>Pain intensity<br>4-pt VRS (0 - none, 1 - mild, 2 - moderate, 3 - severe)<br><br>Pain relief<br>5-pt VRS (0 - no relief, 1 - slight, 2 - moderate, 3 - significant, 4 - complete)<br><br>Ability to perform activities of daily living<br>100mm VAS<br><br>Presence of associated symptoms (0 - absent, 1 - mild, 2 - moderate, 3 - severe)<br><br>Menstrual flow<br>100mm VAS<br><br>Overall analgesic efficacy (insufficient, mediocre, good, excellent)<br><br>Preferred treatment cycle	Dexketoprofen 12.5mg<br>SPID6 (VAS) 196.6 ± 143.9<br>TOTPAR6 15.8 ± 7.4<br>Global good/excellent 74%<br>Time to onset NR<br>Time to remedication 347.4 ± 54.6<br><br>Dexketoprofen 25mg<br>SPID6 199.0 ± 140.7<br>TOTPAR6 15.9 ± 6.9<br>Global good/excellent 82%<br>Time to onset NR<br>Time to remedication 344.0 ± 61.3<br><br>Ketoprofen 50mg<br>SPID6 248.4 ± 135.9<br>TOTPAR6 17.7 ± 5.9<br>Global good/excellent 82%<br>Time to onset NR<br>Time to remedication 353.2 ± 40.9<br><br>Placebo<br>SPID6 (VAS) 89.4 ± 175.8<br>TOTPAR6 8.5 ± 8.2<br>Global good/excellent 32.6%<br>Time to onset NR<br>Time to remedication 288.0 ± 112.2<br><br>No significant differences between the three active treatments, but all significantly better than placebo	Patients remedicating within 1 hr were excluded, after 1 hr LOCF for pain intensity and pain relief set to 0.<br><br>Single dose phase - only patients completing at least the 1st hr post study drug administration in all 4 cycles were included (N=44). Repeated dose phase - only patients taking a minimum of 2 doses of study drug and assessments were available for all cycles (N=13).<br><br>8 patients were excluded; 3 patients were lost to follow-up, 2 patients remedicated within 1 hr, 2 patients failed to comply with the protocol, 1 patient withdrew due to inefficacy<br><br>30 patients reported 45 adverse events, most were mild to moderate, there were no significant differences between groups, no event caused withdrawal. One serious adverse event occurred but not related to treatment	Dexketoprofen 12.5mg<br>No with >1 AE 7<br>All cause withdrawals NR<br>AE withdrawals 0<br><br>Dexketoprofen 25mg<br>No with >1 AE 10<br>All cause withdrawals NR<br>AE withdrawals 0<br><br>Ketoprofen 50mg<br>No with >1 AE 8<br>All cause withdrawals NR<br>AE withdrawals 0<br><br>Placebo<br>No with >1 AE 5<br>All cause withdrawals NR<br>AE withdrawals 0	R 2<br>DB 2<br>WD 1<br><br>Total = 5<br><br>OPVS = 13/16
Mercorio et al. Oral dexketoprofen for pain treatment during diagnostic hysteroscopy in postmenopausal women. Maturitas 2002; 43: 277-281.	R, parallel group<br><br>All patients were referred to outpatient clinic for hyteroscopy due to uterine bleeding.<br><br>Assessed during the procedure, at 30, 60, and 120 mins	Diagnostic hysteroscopy pain<br><br>N= 305<br><br>1 centre in Italy	Dexketoprofen 25mg (1hr before the procedure)<br>N= 148<br><br>Intracervical injection of 5ml mepivacaine 2%<br>N= 150	Pain intensity<br>10cm VAS	Significantly lower pain scores with oral dexketoprofen than intracervical mepiviacaine between 30 and 120 minutes	7 patients were excluded; 5 patients were too anxious to tolerate the procedure, 2 patients due to previous conization. Hysteroscopy was unsuccessful in 10 patients (5 per group) due to pain and for 3 patients in the dexketoprofen due to unsatisfactory view	No data reported	R 2<br>DB 0<br>WD 1<br><br>Total = 3<br><br>OPVS = 7/16

**Limb injury**								
Leman et al. Randomised controlled trial of the onset of analgesic efficacy of dexketoprofen and diclofenac in lower limb injury. Emerg Med J 2003; 20: 511-513.	RCT, DB, single oral dose, parallel groups<br><br>Assessed at baseline, 15, 30, 45, and 60 mins<br><br>Medication administered to patients with acute lower limb injury and a pain score of at least 3, nurse judged whether patient required narcotic analgesia instead	Lower limb injury<br><br>N= 122<br><br>1 centre in UK	Dexketoprofen 25mg<br>N= 65<br><br>Diclofenac 50mg<br>N= 57	Pain intensity<br>11-pt VRS	Dexketoprofen 25mg<br>Pain score baseline 6.35 (5.99 to 6.72)<br>Pain score 15 mins 5.65 (5.2 to 6.09)<br>Pain score 30 mins 4.98 (4.57 to 5.4)<br>Pain score 45 mins 4.51 (4.07 to 4.95)<br>Pain score 60 mins 4.46 (3.98 to 4.93)<br><br>Diclofenac 50mg<br>Pain score baseline 6.33 (6.01 to 6.66) - p=0.5<br>Pain score 15 mins 6.18 (5.85 to 6.50) - p=0.026<br>Pain score 30 mins 5.68 (5.34 to 6.03) - p=0.009<br>Pain score 45 mins 5.4 (5.02 to 5.78) - p=0.002<br>Pain score 60 mins 5.29 (4.88 to 5.7) - p=0.008<br><br>Significantly better pain reduction with dexketoprofen than diclofenac over 60 minutes	8 patients requested further analgesia<br><br>19 patients had either missing data sheets or study drug boxes were found to be empty post randomisation<br><br>No drug-related adverse events were reported	NR	R 2<br>DB 1<br>WD 1<br><br>Total = 4<br><br>OPVS = 10/16
Keller FT. Multi-center, double-blind study to evaluate the efficacy and safety of oral dexketoprofen trometamol in comparison to paracetamol-codeine in the treatment of ankle sprains. Clinical trial report 1999	RCT, DB, three oral doses daily over 4 days, parallel groups<br><br>Assessed at baseline and day 4<br><br>Medication administered to patients with acute distortion of the ankle joint (not requiring surgery or cast) presenting within 24 hrs of injury an pain intensity of (pain on motion) of at least 5cm on 10cm VAS	Ankle sprain<br><br>N= 210<br><br>21 centres in Germany and UK	Dexketoprofen trometamol 25mg TID<br>N= 106<br><br>Paracetamol 500mg ± codeine 30mg TID<br>N= 103	Pain on movement<br>4-pt VRS (1 - absent, 2 - mild, 3 - moderate, 4 - severe)<br><br>Pain at rest<br>4-pt VRS (1 - absent, 2 - mild, 3 - moderate, 4 - severe)<br><br>Pain on pressure<br>4-pt VRS (1 - absent, 2 - mild, 3 - moderate, 4 - severe)<br><br>Pain on movement<br>10cm VAS<br><br>Pain at rest<br>10cm VAS<br><br>Pain on pressure<br>10cm VAS<br><br>Ankle circumference<br><br>Overall efficacy - patient<br>4-pt VRS (none, mediocre, good, excellent)<br><br>Overall efficacy - physician<br>4-pt VRS (none, mediocre, good, excellent)	Dexketoprofen trometamol 25mg TID<br>Pain on movement VAS 7.67 ± 1.13 to 2.74 ± 0.26cm<br>Pain on pressure VAS 7.66 ± 0.18 to 3.02 ± 0.26cm<br>Pain at rest VAS 4.94 ± 0.25 to 1.22 ± 0.19cm<br>Pain on movement (mild/absent) 66<br>Pain on pressure (mild/absent) 68<br>Pain at rest (mild/absent) 96<br>Overall efficacy (patient) good/excellent 80<br>Overall efficacy (physician) good/excellent 88<br><br>Paracetamol 500mg ± codeine 30mg TID<br>Pain on movement VAS 7.91 ± 0.14 to 3.49 ± 0.26cm<br>Pain on pressure VAS 7.83 ± 0.15 to 3.57 ± 0.27cm<br>Pain at rest VAS 5.14 ± 0.15 to 1.38 ± 0.15cm<br>Pain on movement (mild/absent) 60<br>Pain on pressure (mild/absent) 52<br>Pain at rest (mild/absent) 91<br>Overall efficacy (patient) good/excellent 81<br>Overall efficacy (physician) good/excellent 76<br><br>No significant difference between the treatments	1 patient was excluded prior to randomisation due to failing to meeting eligibility criteria, 7 patients withdrew for reasons other than lack of efficacy and were excluded from efficacy analyses<br><br>A total of 12 patients reported 14 adverse events, there were no significant differences between groups and all adverse events were mild in intensity except one case of moderate gastric pain/heartburn. No serious adverse events were reported.	Dexketoprofen trometamol 25mg TID<br>No with >1 AE 5<br>All cause withdrawals<br>AE withdrawals<br><br>Paracetamol 500mg ± codeine 30mg TID<br>No with >1 AE 7<br>All cause withdrawals<br>AE withdrawals	R 2<br>DB 2<br>WD 1<br><br>Total = 5<br><br>OPVS = 13/16

**Bone cancer pain**								
Rodríguez et al. Double-blind evaluation of short-term analgesic efficacy of orally administered dexketoprofen trometamol and ketorolac in bone cancer pain. Pain 2003; 104: 103-110.	R, DB, parallel group, patients treated with continuous and scheduled regimen of opioids or NSAIDs (except acetaminophen and acetylsalicyclic acid) in the previous 15 days were excluded<br><br>Assessed at baseline, day 3, and day 7<br><br>Medication administered when pain intensity at least 40mm on a 100mm VAS at approx every 6hrs for 7 days	Bone cancer pain<br><br>N= 115<br><br>12 centres in Spain	Dexketoprofen trometamol 25mg<br>N= 57<br><br>Ketorolac 10mg<br>N= 58	Pain Intensity<br>100mm VAS<br><br>Pain Intensity<br>4pt-VRS (1 - slight, 2 - bothersome, 3 - severe, 4 - unbearable)<br><br>Pain frequency<br>4pt-VRS (1 - seldom, 2 - frequent, 3 - very frequent, 4 - continuous)<br><br>Analgesics taken<br>4-pt VRS (1 - few, 2 - few but regularly, 3 - a lot and regularly, 4 - a lot and continuously)<br><br>Incapacity due to pain<br>4-pt VRS (1 - autonomous activity, 2 - occasional help, 3 - frequent help, 4 - complete (confined to bed))<br><br>Sleep disturbance<br>4-pt VRS (1 - normal, 2 - wakes up, 3 - insomnia, 4 - use of hypnotics/sedatives)<br><br>Overall efficacy (patient and physician)<br>4-pt VRS (0 - ineffective, 1 - poorly effective, 2 - quite effective, 3 - very effective)	Dexketoprofen tromethamine 25mg<br>PID >20mm from baseline to final visit 31<br>SPID NR<br>TOTPAR NR<br>Global good/excellent NR<br>Time to onset/peak NR<br>Time to remedication NR<br>No remedicating 71%<br><br>Ketorolac 10mg<br>PID >20mm from baseline to final visit 27<br>SPID NR<br>TOTPAR NR<br>Global good/excellent NR<br>Time to onset/peak NR<br>Time to remedication NR<br>No remedicating 72%<br><br>Very little difference between the treatments, with tendency towards dexketoprofen as being better	Remedication was permitted; patients exceeding 1mg paracetamol and 60mg codeine were excluded<br><br>18 patients did not complete the study; 7 due to lack of efficacy, 6 due to adverse events, 5 due to concomitant disease. 2 patients were excluded from efficacy population due to missing efficacy assessments<br><br>Most adverse events were mild or moderate intensity, 3.5% of patients reported serious adverse events in both treatment groups, 6 patients withdrew due to adverse events. There were 3 deaths, none considered related to treatment	Dexketoprofen tromethamine 25mg<br>No with >1 AE 33%<br>All cause withdrawals 5<br>AE withdrawals 1<br><br>Ketorolac 10mg<br>No with >1 AE 35%<br>All cause withdrawals 13<br>AE withdrawals 5	R 2<br>DB 2<br>WD 1<br><br>Total = 5<br><br>OPVS = 13/16

Abbreviations: RCT = randomised controlled trial; R = randomised; DB = double blund; wD = withdrawal or dropout; OPVS = Oxfoprd Pain validity Score; LOCF - last observation carried forward; ITT = intention to treat; N = number; LA = local anaesthetic; VAS = visual analogue scale; VRS = verbal rating scale; AE = adverse event; SPID = summed pain intensity difference; TOTPAR = total pain relief
