# Supplementary material for: Systematic review of dexketoprofen in acute and chronic pain
Source: BMC Clin Pharmacol. 2008 Oct 31;8:11. doi: 10.1186/1472-6904-8-11 (PMC2585070; doi:10.1186/1472-6904-8-11)
Supplement: Additional file 6 — Trials of oral dexktoprofen in arthritis. The file contains information on each included study, with reference, quality score, design, treatments, main results, and comments. [file 1472-6904-8-11-S6.pdf]

Additional file 6: Trials of oral dexktoprofen in arthritis

Reference	Methods	Details	Dosing regimen	Outcome measures	Efficacy results	Remedication, exclusions, and adverse events	Safety results	Quality score
Beltrán et al. Comparison of dexketoprofen trometamol and ketoprofen in the treatment of osteoarthritis of the knee. J Clin Pharmacol 1998; 38 (12 Suppl):74S-80S.	RCT, DB, 3 daily oral doses over 3 wks, parallel groups, 7-15 day analgesic washout<br><br>Assessed pre-washout, at baseline and at weekly intervals<br><br>OA knee pain of at least 3 month's duration, regular NSAID use, Lequesne severity index of 5 to 13, and a Kellgren and Lawrence radiologic grade of 2-4, no mention of baseline pain intensity but baseline demographics indicate it was severe	OA knee<br><br>N= 183<br><br>11 centres in Spain	Dexketoprofen trometamol 25mg TID<br>N= 89<br><br>Ketoprofen 50mg TID<br>N= 94	Pain intensity<br>100mm VAS<br><br>Lequesne index (functional ability)<br><br>Patient overall assessment of symptoms<br>(much worse, worse, a little worse, same, a little better, better, much better)<br><br>Physician overall assessment of symptoms<br>(much worse, worse, a little worse, same, a little better, better, much better)	Dexketoprofen trometamol 25mg TID<br>Pain intensity (baseline) 63.9 + 22.6<br>Pain intensity (wk 1) 38.6 + 25.4<br>Pain intensity (wk 3) 32.1 + 25.6<br>Lequesne index (wk 1) 8.5 (6-11)<br>Lequesne index (wk 3) 7 (5-10)<br>Physician overall (wk 3) 73.2% better/much better<br>Patient overall (wk 3) 67.1% better/much better<br><br>Ketoprofen 50mg TID<br>Pain intensity (baseline) 63.2 + 20.9<br>Pain intensity (wk 1) 40.2 + 25.6<br>Pain intensity (wk 3) 40.5 + 28.2<br>Lequesne index (wk 1) 8 (6-10)<br>Lequesne index (wk 3) 8 (6.5-11)<br>Physician overall (wk 3) 50% better/much better<br>Patient overall (wk 3) 45.5% better/much better<br><br>Significant reduction in pain at three weeks for dexketoprofen over ketoprofen	Remedication permitted, LOCF used where patients discontinued early<br><br>3 patients were excluded from analyses, 2 (1 per group) were lost to follow up and 1 patient in the dexketoprofen group did not take medication<br><br>A total of 65 patients reported 101 adverse events, there were no significant differences between groups, and the majority of events were mild or moderate in severity, no serious adverse events were reported	Dexketoprofen trometamol 25mg TID<br>No with >1 AE 27<br>All cause withdrawals 6<br>AE withdrawals 5<br><br>Ketoprofen 50mg TID<br>No with >1 AE 38<br>All cause withdrawals 11<br>AE withdrawals 7	R 2<br>DB 2<br>WD 1<br><br>Total = 5<br><br>OPVS = 13/16
Marenco et al. A multicentre, randomised, double-blind study to compare the efficacy and tolerability of dexketoprofen trometamol vs diclofenac in the symptomatic treatment of knee osteoarthritis. Clin Drug Invest 2000; 19: 247-256.	RCT, DB, 3 daily oral doses over 2 wks, parallel groups, 7-14 day analgesic washout<br><br>Assessed pre-washout, at baseline and at weekly intervals<br><br>OA knee pain of at least 3 month's duration, regular NSAID use, Lequesne severity index of 5 to 13, and a Kellgren and Lawrence radiologic grade of 2-4, persistent pain during washout period	OA knee<br><br>N= 117<br><br>8 centres in Spain	Dexketoprofen trometamol 25mg TID<br>N= 63<br><br>Diclofenac 50mg TID<br>N= 54	Pain intensity<br>100mm VAS<br><br>Pain intensity<br>4-pt VRS (no pain, mild, moderate, severe) - 1st wk only<br><br>Lequesne index (functional ability)<br><br>Patient overall assessment of symptoms<br>(much worse, worse, a little worse, same, a little better, better, much better)<br><br>Physician overall assessment of symptoms<br>(much worse, worse, a little worse, same, a little better, better, much better)	Dexketoprofen trometamol 25mg TID<br>Pain intensity (baseline) 61.7 + 18.5<br>Pain intensity (wk 1) 45.4 + 24.9<br>Pain intensity (wk 2) 34.7 + 22.3<br>Lequesne index (wk 1) 9 (6-11)<br>Lequesne index (wk 2) 8 (6-10)<br>Physician overall (wk 2) 31.2% little better<br>Patient overall (wk 2) 36.1% little better<br><br>Diclofenac 50mg TID<br>Pain intensity (baseline) 62.1 + 22.8<br>Pain intensity (wk 1) 43.3 + 22.9<br>Pain intensity (wk 2) 40.6 + 22.2<br>Lequesne index (wk 1) 8.5 (7-11.5)<br>Lequesne index (wk 2) 8.5 (6-10.5)<br>Physician overall (wk 2) 27.8% little better<br>Patient overall (wk 2) 25.9% little better<br><br>No significant difference between treatments	Remedication permitted, LOCF used where patients discontinued early<br><br>2 patients were excluded from the ITT analysis as they were lost to follow-up<br><br>A total of 37 patients reported 63 adverse events, there were no significant differences between groups, and the majority of events were mild or moderate in severity, no serious adverse events were reported	Dexketoprofen trometamol 25mg TID<br>No with >1 AE 19<br>All cause withdrawals 9<br>AE withdrawals 6<br><br>Diclofenac 50mg TID<br>No with >1 AE 18<br>All cause withdrawals 9<br>AE withdrawals 6	R 2<br>DB 2<br>WD 1<br><br>Total = 5<br><br>OPVS = 13/16

Abbreviations: RCT = randomised controlled trial; R = randomised; DB = double blund; wD = withdrawal or dropout; OPVS = Oxfoprd Pain validity Score; LOCF - last observation carried forward; ITT = intention to treat; N = number; LA = local anaesthetic; VAS = visual analogue scale; VRS = verbal rating scale; AE = adverse event; SPID = summed pain intensity difference; TOTPAR = total pain relief
